# Genomes of N_2_-fixing endosymbionts of unicellular eukaryotes and host-independence

**DOI:** 10.1186/s12864-026-12517-0

**Published:** 2026-02-05

**Authors:** Jeff Elhai

**Affiliations:** Richmond, USA

**Keywords:** Nitrogen fixation, Symbiosis, Endosymbiosis, Metabolism, Carbon transport, Nitrogen transport, Evolution

## Abstract

**Background:**

The projected 2.7-fold increase in population in sub-Saharan Africa by the end of the century demands consideration as to how agricultural output can keep pace. Augmenting nitrogen inputs is a practical necessity, but this must be accomplished in such a way that avoids the environmental costs of past advances and also places the resource in the hands of those who will be the most affected. Biological nitrogen fixation might play an important role. The realization that certain algae are able to provide for their own nitrogen needs by fixing atmospheric N_2_ raises the possibility that an endosymbiont responsible for the nitrogen might be transferred to crop plants. For this to take place, it is necessary that the endosymbionts be (or be made to be) sufficiently independent of their hosts so that they may establish themselves in crop plants appropriate to African agriculture.

**Results:**

Genomes from six endosymbionts from diatoms within the family Rhopalodiaceae were analyzed. They were compared to genomes from free-living cyanobacteria and to those of the nitroplast UCYN-A and chromatophore from Paulinella, to which they are related. Unlike the latter two endosymbionts, the six from Rhopalodia encode all the enzymes considered that underlie metabolic processes and provide the energy to power N-fixation. Some of the endosymbionts also appear able to synthesize cofactors essential for central metabolism. The analysis points to possible carbon sources the endosymbionts might take up from their hosts, including glycerol and chitobiose. Possible routes of nitrogen export to the host were also examined.

**Conclusions:**

Within the limits of genome analysis, some of the Rhopalodian endosymbionts appear to be metabolically independent of their hosts, except for requiring a carbon source. However, the choice of carbon source and the likely means of nitrogen export are not compatible with crop plants. Genetic modification would surely be necessary for any prospect of propagation of an endosymbiont in a plant of agricultural importance, and significant questions must first be answered in the laboratory. To this end, the endosymbiont of *Epithemia clementina* may be best suited for such investigations, eventually after transfer to the model diatom *Phaeodactyllum tricornutum.*

**Supplementary Information:**

The online version contains supplementary material available at 10.1186/s12864-026-12517-0.

## Background

For the past 60 years, the increase in global agricultural production has depended on a concomitant increase in the application of nitrogenous fertilizer [1, 2]. This has come at a cost, both economic [3] and environmental [4, 5], and climate change promises to make these costs even greater [6]. Historically, the price of fertilizer has been too great for the majority of farmers in sub-Saharan Africa [7, 8], where food needs are most intense and which will contribute most of the population rise in the coming decades [9]. To keep pace, it would seem that the world must find a way to increase the production of nitrogenous fertilizer, especially in sub-Saharan Africa, but that would also increase the substantial harm that accompanies its use. While this may seem like a global problem, it is best viewed as local, addressed by technologies that can adapt to the great heterogeneity confronting African farmers and that can take advantage of input provided by the farmers themselves [8].

In principle, biological nitrogen fixation offers a way out. N_2_-fixing rhizobia within specialized root nodules of legumes meet the nitrogen needs of their plant hosts in a way that’s low cost and environmentally benign. However, the interactions between rhizobia and their hosts are famously complex [10], and we remain a long way from extending the benefits of root nodule N_2_-fixation to crop plants beyond the legumes [11]. Other approaches have been explored – inoculation with associative N_2_-fixing bacteria, engineering plants to express nitrogenase and related genes, changes in agronomic practices – each with its own problems [12–14].

An alternate strategy has not been given much attention, inspired by the example of the chloroplast. Chloroplasts reduce atmospheric CO_2_ to sugar for use by the plant. Why not an organelle that reduces abundant atmospheric N_2_ to ammonia? [15, 16]. This organelle – call it a nitroplast – might provide for the nitrogen needs of diverse crop plants, just as chloroplasts provide for their carbon needs. This may sound like the stuff of science fiction, but such organelles already exist! [17]. The haptophyte alga *Braarudosphaera bigelowii* contains an endosymbiont (called UCYN‑A), phylogenetically related to unicellular cyanobacteria, that provides its algal host fixed nitrogen from N_2_. Its division is integrated with the cell division cycle of its host. It is everything one could ask for in a nitroplast with agricultural potential…

…except for the ability to function in a broad range of hosts. Even chloroplasts within the same species may exhibit different levels of compatibility with different genomes [18], although successful transfer of chloroplasts to members of different closely related species has been demonstrated [19]. A narrow range of compatibility is expected because the encoding of chloroplast proteins is split between the nucleus and the chloroplast, and the proteins encoded by the nucleus of one species may not entirely match the proteins needed by a chloroplast of another. In addition, multiprotein complexes may not work when mixing protein components or regulatory mechanisms of different species [18, 20]. The nitroplast UCYN-A could not possibly function in crop plants. Like chloroplasts, it imports hundreds of proteins from its host, many required for basic functions [17]. It stretches the limits of credulity to think that a land plant could have the ability to replace these proteins, even if the protein import systems [21] of the old and new hosts were compatible.

Now consider the nitroplast-like endosymbionts (also called spheroid bodies) found within diatoms of the family Rhopalodiaceae [22, 23]. They too are related to cyanobacteria, provide nitrogen to their hosts [24], and appear to be stably inherited [25, 26]. However, they are much younger than the nitroplast of *B. bigelowii* (UCYN-A), initially acquired by a diatom an estimated 12 to 34 million years ago [27, 28], compared to the 100 million years nitroplasts in *B. bigelowii* have been around [29] (and the more than 1.6 billion years since the acquisition of the chloroplast [30]). One would expect that diatom nitroplasts have not progressed as much in the integration of organelle and host. If the diatom nitroplasts are sufficiently host-independent, it is conceivable that they could be transferred to a new host, perhaps even to a crop plant.

Consider also our seemingly favored moment in the course of evolutionary history. Chloroplasts evidently arose from a single acquisition of an ancient cyanobacterium. A second acquisition led to the chromophore of Paulinella a 100 million years ago. UCYN-A represents a third acquisition – three cyanobacterial endosymbioses over the course of 1.6 billion years. Yet we are witness to multiple acquisitions in relatively recent times – at least one cyanobacterium by a Rhopalodian diatom, another by a diatom within the genus Climacodium [31], a third of a heterocyst-forming cyanobacterium (the ancestor of *Richelia euintercellularis*) by a diatom within the genus Hemiaulus [32], and another of a marine Synechococcus by a dinoflagellate [33]. Perhaps it is just luck that we can observe four rare events in evolution that happen to have occurred not so distant from today.

Alternatively, it is possible that there is no favored time, that cyanobacterial acquisition is common [33], just as is acquisition of heterotrophic bacteria by insects and other eukaryotes [34], but they generally don’t persist. Except for a few rare cases, they come and go. In that case, the recent acquisitions give us an opportunity to investigate why the ancestors of these endosymbionts were successful, at least for the moment. What cyanobacterial characteristics are favorable to endosymbiosis that persist at least as far as they have?

Genomes have now been sequenced from at least six endosymbionts from Rhopalodian diatoms [24, 35–38]. Each genome is much reduced from the genomes of related free-living cyanobacteria, 2.5 Mb to 3.1 Mb for the endosymbionts compared to about 5 Mb for their closest cyanobacterial relatives [39, 40]. The availability of these genomes makes it possible to assess what are the metabolic capabilities of the endosymbionts, to what degree they are dependent on their diatom hosts, and what may be required for a successful transfer to a crop plant, without need to otherwise modify the plant. Furthermore, if each endosymbiont has to some extent lost genes independently from the others, then a comparison of their genomes offers the prospect of assessing what common genes may be under selection within the endosymbiotic environment.

## Results

### Genome characteristics and phylogeny of the nitrogen-fixing endosymbionts

**Fig. 1:**
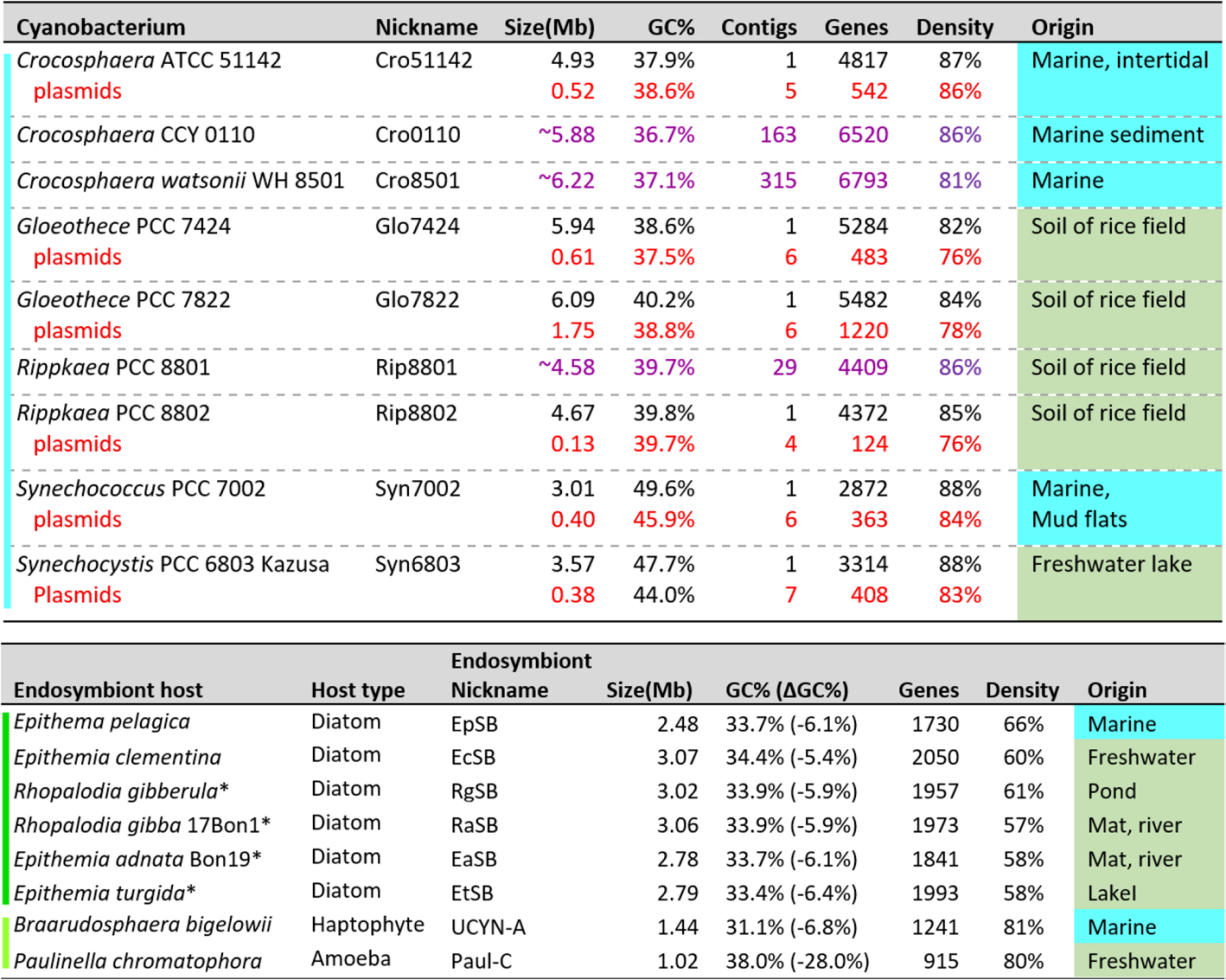
Genomes used in this study. Genome sources are given for the six Rhopalodian (diatom) endosymbionts central to this study (dark green bar), along with those of related free-living cyanobacteria (cyan bar). The genomes of two other cyanobacteria-derived endosymbionts are also listed (yellow-green bar). When the organism has plasmids in addition to the chromosome, information related to the chromosome is given in black and that related to the plasmids (taken as a whole) is given in red. In the three cases where the genome sequence is incomplete, no distinction can be made between chromosome and plasmid, and information is given in purple. Four endosymbionts (marked by asterisks) have small plasmids which have not been considered in this study. The gene density was calculated for a given entity by dividing the sum of nucleotides participating in coding and non-coding genes by the number of nucleotides in that entity or entities. See Supplemental Table S1 for sources

Before comparing the genomes of the endosymbionts to gain insight into the endosymbiotic state, it is important to see how the genomes are related to each other and to those of free-living cyanobacteria (see Fig. 1 and Supplemental Table S1 for the genomes considered in this study and their abbreviations). One shouldn’t place too much stock in the names of the host diatoms – Epithemia and Rhopalodia. The two genera are both paraphyletic, with species of one intermixed with the other in phylogenetic trees [37, 41]. For this reason, I’ve lumped them together as ‘Rhopalodian’.

**Fig. 2:**
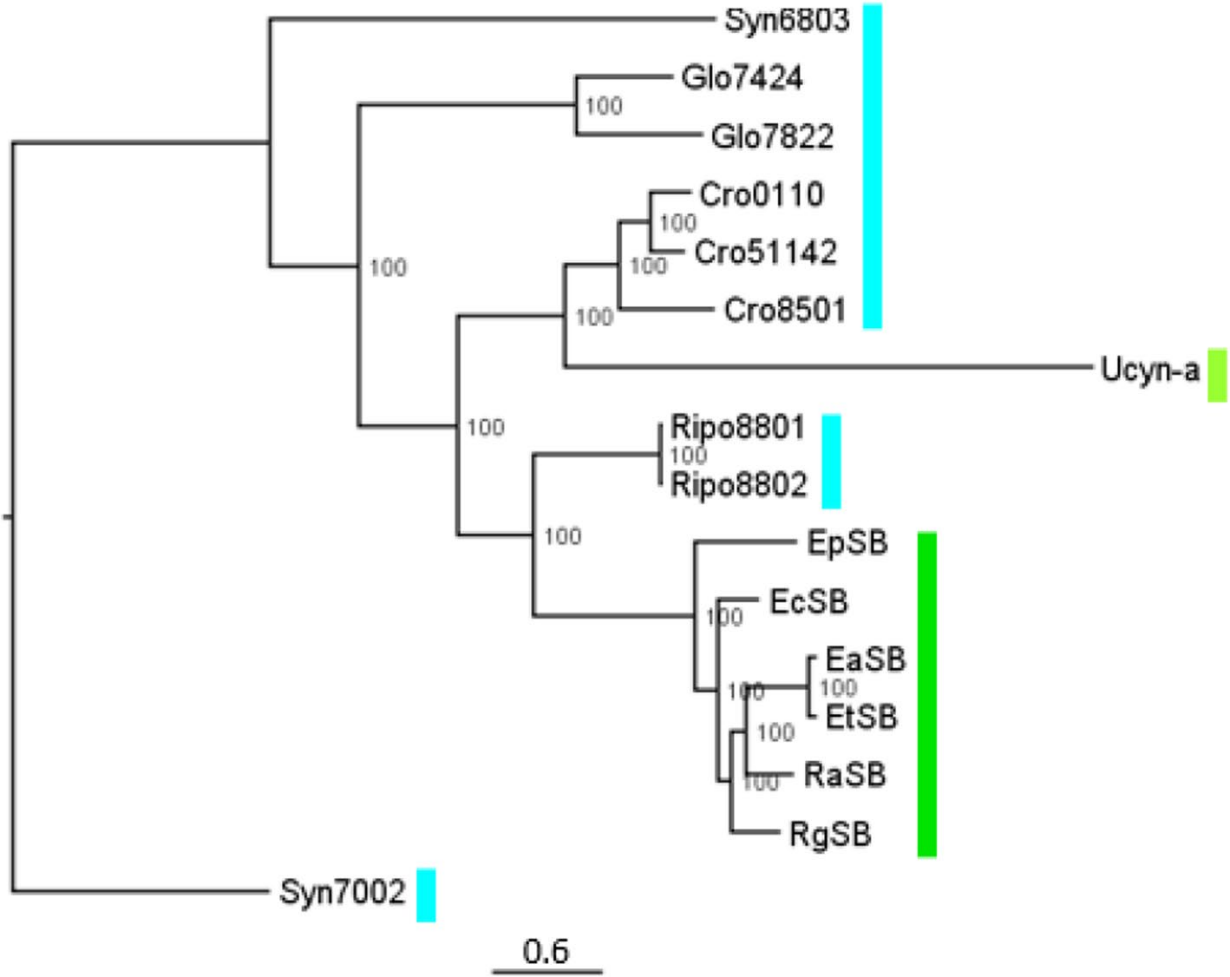
Phylogenetic relationship amongst Rhopalodian endosymbionts and close relatives. The tree was based on the sequences of 29 highly conserved protiens (see Materials and Methods Supplementary Table S5). The dark green bar indicates the Rhopalodian endosymbionts, the yellow-green bar indicates non-Rhopalodian endosymbiont, and the cyan bar indicates free living cyanbacteria. The numbers at nodes indicates percent bootstrap support in 100 repetitions. The scale bar represents the number of inferred substitutions per site

Figure 2 shows a phylogeny based on 29 proteins with orthologs encoded in the genomes of all endosymbionts and closely related free-living cyanobacteria. It is concordant with trees based on hundreds of proteins [38, 42] but discordant with trees based on just the NifH protein plus 16S rRNA [24, 37]. All the Rhopalodian endosymbionts cluster together, with *Rippkaea* PCC 8801 (Rip8801) and PCC 8802 (Rip8802), the most closely related free-living cyanobacteria. They are separated from the well-studied nitroplast from *B*. *bigelowii*, UCYN-A, which is more closely related to members of the cyanobacterial genus Crocosphaera. The tree implies that there were at least two primary acquisitions of cyanobacteria to form N_2_-fixing endosymbionts – one leading to UCYN-A and its close relatives [43] and at least one leading to the Rhopalodian endosymbionts.

Four of the six Rhopalodian endosymbiont genomes (EaSB, EtSB, RaSB, RgSB) are closely related to each other (Fig. 2). The genome most distant within this group, that of EpSB, comes from the lone marine diatom in the set of six, raising the possibility that the association might have arisen from a separate acquisition of a cyanobacterium similar to the progenitor of the other Rhopalodian endosymbionts. This idea is explored in the Discussion section.

The sizes of the genomes of the Rhopalodian endosymbionts range from 2.5 to 3.1 Mb, compared to the 4.6 Mb size of the genome of its closest free-living relative and the 1.4 Mb size of the genome of the non-Rhopalodian endosymbiont, UCYN-A (Fig. 1A). All of their genomes exhibit a decrease in GC% relative to their closest free-living relative. Both size reduction and reduced GC fraction are typical of obligate endosymbionts [34, 44]. In four cases (EaSB, EtSB, RaSB, RgSB), a small (~ 6 kb) plasmid was reported in addition to the chromosome. All four plasmids have a high degree of identity and apparently share the same five genes [38]. It isn’t known whether the remaining endosymbiont genomes (EcSB, EpSB) have plasmids.

**Fig. 3:**
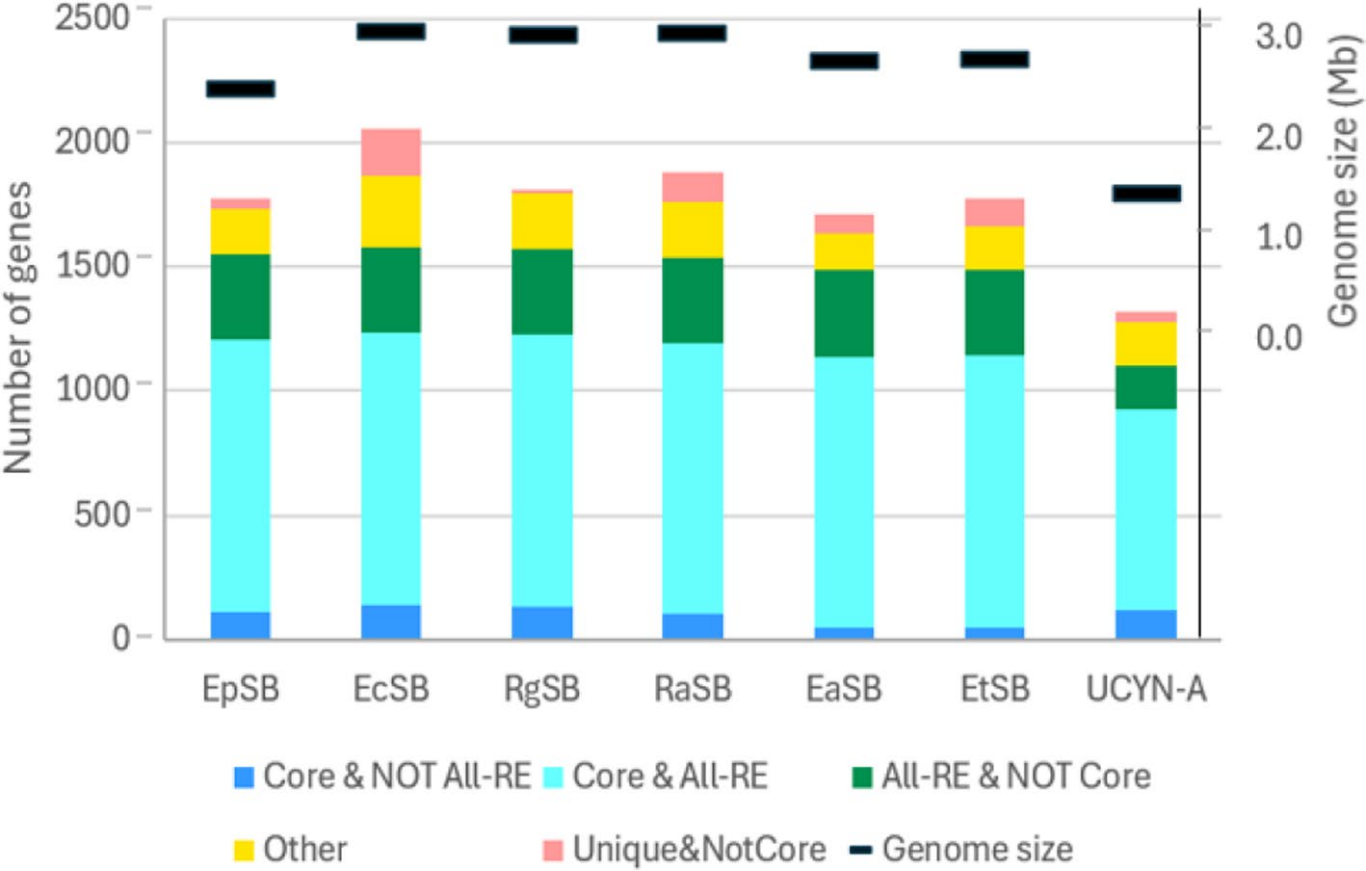
Fraction of protein-encoding genes in different sets. For each endosymbiont genome, the numbers of protein-encoding genes in the indicated classes are stacked on top of each other. For comparison, the size of each genome is also shown, on a different scale. See Fig. 1 for abbreviations of the endosymbionts and genome sizes. See Supplemental Table S3A for counts of genes in each category

However, the difference in genome sizes amongst the Rhopalodian endosymbionts is deceptive. Supplemental Table S2 A lists the proteins and orthologous relationships of all the endosymbionts and Rip8802 and *Crocosphaera* ATCC 51142 (Cro51142). Proteins that are conserved amongst all of the close relatives of the endosymbionts (Fig. 1), called here the *core* proteins (1779 proteins, indicated in Supplemental Table S2A, column "Core?"), appear in the endosymbiont genomes in approximately the same numbers, somewhat less in EtSB and EaSB (Fig. 3, Supplemental Table S3A). The apparent difference in protein number amongst the endosymbionts is explained by differences in the numbers of non-conserved proteins, particularly proteins unique to a genome, which are overwhelmingly biased towards small proteins (< 100 amino acids). This characteristic suggests that much of the differences in the number of proteins amongst the endosymbionts may be artifactual, attributable to differences in gene-calling programs and their variable tolerance to small sizes. The differences in the genome sizes are probably better interpreted not as differences in coding capability but rather the degree to which broken genes (pseudogenes) have remained in the genome. The gene density my serve as a proxy for the removal of pseudogenes. This measure is high for UCYN-A, intermediate for EpSB, and low for the other Rhopalodian endosymbionts (Fig. 1). The significance of gene density is considered later.

The availability of a set of core proteins makes it possible to gain insight into what conserved proteins that were almost surely present in the free-living ancestor(s) of the Rhopalodian endosymbionts proved to be unnecessary in the symbiotic state and disappeared. To address this issue, a second set, *No-RE*, was defined: those proteins found in free-living relative Rip8802 with no ortholog in any Rhopalodian endosymbiont (Supplemental Table S2A, column "status"). Its intersection with core proteins is given in Supplemental Table S3B, with highlights in Table 1. Some on the list are to be expected. The endosymbionts have lost the ability to fix carbon, so it is no surprise to see the absence of proteins related to Photosystems I and II, antenna, carboxysomes, and carbon fixation. Many of the other entries suggest loss of redundancies, e.g. loss of a conserved copy of dihydroorotate dehydrogenase (pyrimidine metabolism). Some of the other entries on the list are more interesting and will be discussed later in context.

**Table 1:** Proteins in Core set but not in Rhopalodian endosymbionts^a^

Description	Number of proteins
Carbon fixation/photorespiration	8
Carboxysome proteins	12
Chlorophyll biosynthesis	2
Cofactor biosynthesis—biotin	1
Cofactor biosynthesis—dihydrofolate	1
Competence-related proteins	4
Cyanophycin metabolism	2
External polysaccharide biosynthesis	14
Glyceraldehyde 3-phosphate dehydrogenase (NAD(P) +, phosphorylating)	1
Hydrogenase (reversible)	6
Nitrate utilization	6
Photosystem I	11
Photosystem II	24
Phycobiliprotein	16
Phylloquinone biosynthesis	7
Pili	7
Superoxide dismutase (Fe-dependent)	1
Tocopherol biosynthesis	3
Tricarboxylic acid cycle	3
Transport proteins	30
Others	348

^a^See Supplemental Table S3B for list of specific proteins

Another useful set of proteins are those that are found in all Rhopalodian endosymbionts (*All-RE*) (Supplemental Table S2A, column "status"). One would expect that most proteins required for the symbiotic state would be contained in this group of 1436 proteins. The great majority of proteins encoded by the Rhopalodian endosymbionts are in this set (Supplemental Table S3A). Most of these are also in the Core proteins (Fig. 3), but those that are not Core proteins are of special interest, because they may point to special characteristics of the ancestor(s) of the endosymbionts that set it apart and enabled it to be successful in the association with its diatom host. Table 2 shows some of the proteins that are in All-RE but are not in Rip8801 and Rip8802, the closest relatives of the endosymbionts (the complete list of 39 such proteins is provided in Supplemental Table S3C). Most are found in one or more of the more distantly related Crocosphaera. Many of these proteins will be discussed individually in the sections that follow.

**Table 2:** Proteins in all Rhopalodian endosymbionts but not in *Rippkaea* PCC 8802^a^

Prototype^b^	Description	In Crocosphaera^c^	Saline habitat^d^
RGRSB-1038	alpha-glucosidase	2	94%
RGRSB-0116	alpha-glucosidase (probable)	2	94%
RGRSB-1815	cytochrome c oxidase (ARTO) subunit I	3	29%
RGRSB-1816	cytochrome c oxidase (ARTO) subunit II	3	31%
RGRSB-1814	cytochrome c oxidase (ARTO) subunit III	3	31%
RGRSB-0743	glucosylglycerol-phosphate phosphatase	2	80%
RGRSB-0176	glucosylglycerol-phosphate synthase	2	73%
RGRSB-0175	glycerol-3-phosphate dehydrogenase (FAD)	2	67%
RGRSB-0112	glyceraldehyde 3-phosphate dehydrogenase (NADP, nonphosphorylating), GapN	1	80%
RGRSB-0442	Superoxide dismutase (Ni-dependent)	1	90%
RGRSB-0441	Superoxide dismutase (Ni-dependent) maturation protein	1	76%
RGRSB-1023	Transport: calcium/sodium antiporter	3	62%
RGRSB-0595	Transport: putative Na +/K +/2Cl- cotransporter	3	77%
RGRSB-0697	Transport: sodium-coupled permease	0	86%
RGRSB-0104	Transport: SulP family inorganic anion transporter	3	70%

^a^See Supplemental Table S3C for full list of Rhopalodian orthologs and more granular habitat information

^b^Protein from the endosymbiont of Rhopalodia gibberula is orthologous to proteins from the other endosymbionts

^c^Count of orthologs in Crocosphaera ATCC 51142, CCY 0110, and WH 8501

^d^Fraction of 127 cyanobacteria with orthologs to the given prototype protein, with habitats listed as {“marine”, “saline lake”, “coastal”, “brackish”, “intertidal”, or “estuary”} and not {“freshwater”, “bog”, “hot springs”, “halo-intolerant”, “terrestrial”, “rock”, “paddy”, “sand”, “soil”, or “root”}

Most proteins within All-RE have orthologs in cyanobacteria heavily biased towards those isolated from saline environments (Table 2 and Supplemental Table S3C). Except for alternative cytochrome oxidase, between 62 to 94% of the orthologs of the proteins are from halophilic cyanobacteria. This is remarkable, because the 127 cyanobacteria considered are overwhelmingly terrestrial and freshwater. Only 34% come from saline environments, only 20% if one focuses on the half of the genomes most closely related to the endosymbionts (thereby excluding the large number of genomes from the marine picocyanobacteria).

### Metabolic analysis – Informational proteins

**Fig. 4:**
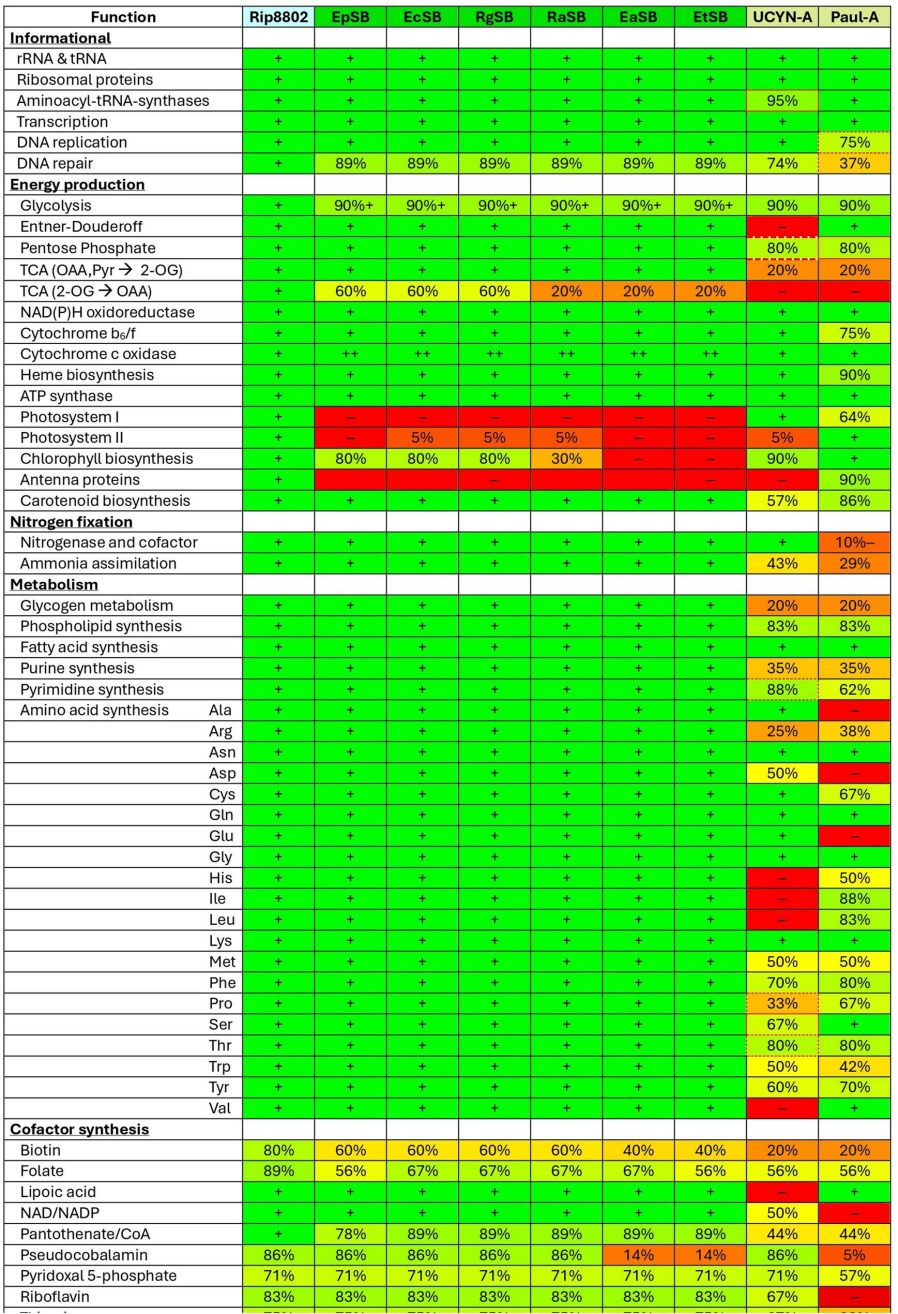
Summary of metabolic capabilities of endosymbionts and related cyanobacterium.Green represents presence of pathway, red indicates absence, and intermediate colors indicate presence of some but not all enzymes and initial substrates. Percentages indicate the fraction of the reactions in a pathway whose enzymes are recognizable in the genome. Lists of the specific enzymes used in the calculation of percentages are given in Supplemental Table S2. If the percentage is followed by a “+” (or a cell contains an extra “+”), then the indicated pathway is supplemented with a capability that goes beyond that possessed by R8802. A cell surrounded by a red outline indicates that the hole in the pathway was filled by importing a protein encoded by the nucleus of Braarudosphaera bigelowii [17] or Paulinella chromatophore [170]. If the outline is dashed, then only one of multiple holes was filled. Organism/endosymbiont abbreviations are explained in Fig. 1.

Any endosymbiont needs to maintain its ability to propagate itself. Figure 4 shows graphically that this minimal bar is likely met by the Rhopalodian endosymbionts without need for additional proteins from their hosts (details given in Supplemental Table S2B). The Rhopalodian endosymbionts encode all the required ribosomal proteins, machinery for transcription, and components for DNA replication. They have retained all but three of 27 proteins related to DNA repair [45], even though such proteins are frequently lost in endosymbiosis [16], most notably in many endosymbionts of insects [46]. In two of those three cases, the function of the missing protein may be assumed by a different protein. In one of them, the missing photolyase PhrB, has the same activity as another photolyase, PhrA, that is found in the endosymbionts. The former has been shown to be of little functional importance in *Synechocystis* [47]. In the second case, RNase HII (absent in all endosymbionts) overlaps in function with RNase HI [48, 49] (present in all endosymbionts).

The situation is somewhat different with UCYN-A, which lacks a protein, methionyl-tRNA-formyltransferase, that is nearly universal protein amongst prokaryotes [50, 51]. The endosymbiont survives because the protein is one of many imported from its host. Similarly, the chromatophore from Paulinella imports from its host the essential DNA replication protein DNA ligase. UCYN-A also lacks seven DNA repair enzymes including the three also absent in the Rhopalodian endosymbionts.

### Metabolic analysis – Energy production

#### Context and lessons learned from free-living cyanobacteria

The Richelia/diatom symbiosis devotes 22% of photosynthetic production by the host to support the endosymbiont [32], and it is likely that the same is true for the Rhopalodia. Apart from the general costs of maintaining existence – e.g. ATP to aminoacylate tRNAs for translation and NADPH for the synthesis of membrane lipids – the Rhopalodian endosymbionts bear the added burden of meeting the considerable energy needs of N_2_-fixation. The process of fixing a single molecule of N_2_ to two molecules of NH_3_ requires 16 ATPs and 8 high-energy electrons [52]. Their production almost surely takes place within the endosymbiont, and metabolic processes must be present to allow that to happen.

In addition, there is the cost of protecting the enzymes of nitrogen fixation from irreversible inactivation by oxygen [53]. Cyanobacteria that form specialized N_2_-fixing cells (called heterocysts) protect nitrogenase from inactivation by ambient oxygen and oxygen produced by photosynthesis in adjacent cells through a variety of measures [54]. Heterocysts have limited permeability to gases, [55] and the sites where gases can enter – the poles of the cells – are protected by a high concentration of cytochrome oxidase [53, 56]. Oxygen is also consumed in a light-dependent fashion [57] by a flavoprotein called Flv3, [58] and by an uptake hydrogenase that redirects the electrons of the H_2_ produced by nitrogen fixation into the electron transport chain [59, 60]. This enables heterocysts to fix nitrogen during the day using photosynthate from vegetative cells and during the night using photosynthate stored as glycogen [61].

Single-cell cyanobacteria closely related to Rhopalodian endosymbionts address the oxygen problem differently, fixing nitrogen only at night, [62] at the expense of glycogen accumulated during the day from photosynthesis [61, 63]. Respiration may play a role in reducing the level of ambient oxygen to a level low enough to permit nitrogen fixation [64].

A consideration of how heterocyst-forming and N_2_-fixing unicellular cyanobacteria address these problems may help in guiding us through the genomes of endosymbionts towards an understanding of their strategies. Heterocyst-forming cyanobacteria when fixing N_2_ during the day can use Photosystem I (PSI)-dependent cyclic photophosphorylation to drive ATP production [65, 66]. They are still able to fix N_2_ in the dark, albeit at a significantly reduced rate, so long as oxygen is present [67], presumably required for oxidative phosphorylation. That process appears to be essential for ATP production in nitrogen-fixing unicellular cyanobacteria as well [62, 68].

NADPH is necessary to reduce (directly or indirectly) the ferredoxin used by nitrogenase as the electron donor. In heterocyst-forming cyanobacteria, NADPH is produced primarily through the pentose phosphate pathway [64]. In Cro51142, the genes encoding enzymes in the pentose phosphate pathway are highly expressed at the onset of darkness, preceding peak nitrogenase expression [69], consistent with a central role in supporting nitrogen fixation.

#### Energy production and oxygen protection in endosymbionts

**Fig. 5:**
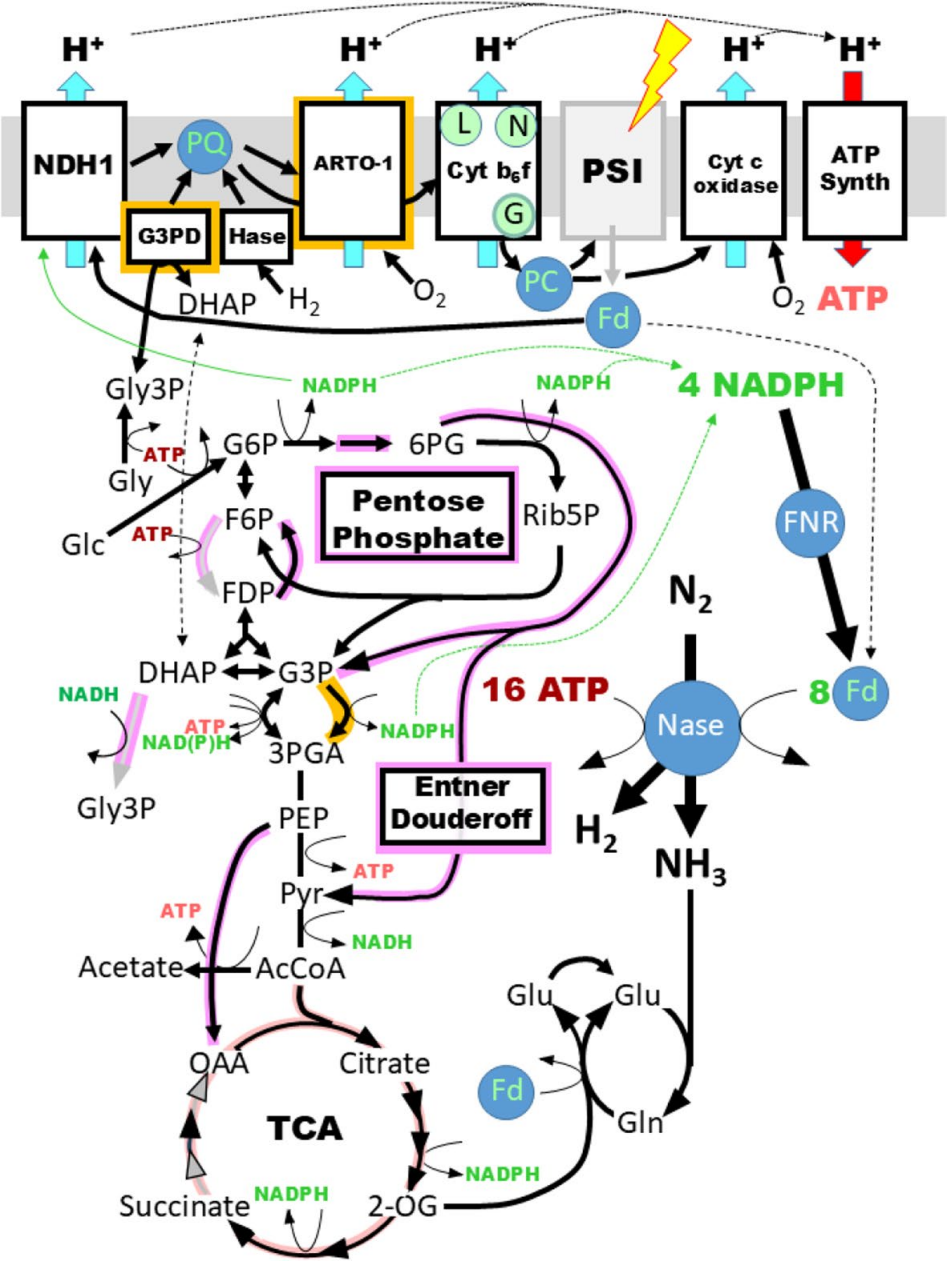
Energy production and consumption in endosymbionts. Reactions present in the Rhopalodian endosymbionts are shown as black lines and arrows. Those absent are shown as gray lines and arrows. Those absent specifically in UCYN-A are highlighted in pink. The production and consumption of ATP is shown in light and dark red, respectively. Electron-carrying proteins are shown as blue circles with green letters. The energy-generating thylakoid membrane, internal to the endosymbionts and to cyanobacteria, is shown as a gray rectangle. Reactions highlighted in orange are found in Rhopalodian nitroplasts but not in their closest cyanobacterial relatives. Pathway maps, abbreviations, specific genes, and justifications are given in Supplemental Table S2

An overview of the metabolic capabilities of the endosymbionts is presented in Fig. 4 (with maps in Supplemental Figs. S01-S08), and Fig. 5 shows a representation of part of central metabolism. While UCYN-A fixes nitrogen solely during daylight hours [70] and lacks enzymes for glycogen metabolism, all Rhopalodian endosymbionts have the coding capacity for enzymes of glycogen metabolism (Supplemental Table S2C and Fig. S01), and at least some fix in daylight and to some extent at night. EpSB fixes mostly but not exclusively during the day, and EcSB fixes continuously without regard to light [22, 24, 37].

The time at which nitrogen fixation takes place is important, because it affects the possibilities of how the ATP required for nitrogen-fixation is produced (Fig. 5 and Supplemental Table S2D). UCYN-A has retained PSI proteins and therefore, like heterocysts, can use cyclic photophosphorylation to produce ATP. The Rhopalodian endosymbionts have lost PSI proteins and must rely primarily on oxidative phosphorylation. Glycolysis is not available even for a minor contribution to the level of ATP because all endosymbionts lack the ability to encode the central enzyme phosphofructokinase (Supplemental Fig. S02). The same is true in many free-living cyanobacteria [71]. However, the Entner-Doudoroff pathway is an alternate route to take glucose-6-phosphate to pyruvate, one that is probably of greater quantitative importance in cyanobacteria than glycolysis [70, 72]. This pathway is present in all Rhopaladian endosymbionts (but not in UCYN-A) (Supplemental Table S2D and Fig. S03).

The tricarboxylic acid cycle is present only partially in the Rhopalodian endosymbionts – from oxaloacetate to 2-oxoglutarate – and from there to succinate in RgSB, EcSB, and EpSB (Fig. 5 and Supplemental Table S2D and Fig. S04). It is entirely absent in UCYN-A. The primary purpose of the pathway is probably to produce the 2-oxoglutarate required by glutamine synthetase/GOGAT, the key enzymes of ammonia assimilation [73]. However, the reductant produced by the partial tricarboxylic acid cycle may also contribute to ATP production by oxidative phosphorylation.

The pentose phosphate pathway is intact in all the endosymbionts (Fig. 5 and Supplemental Table S2D and Fig. S03). This includes UCYN-A, which does not encode its own 6-phosphogluconolactonase but imports it from its host [17]. The expression of one central enzyme (glucose-6-phosphate dehydrogenase) has been measured in one endosymbiont and found to be extraordinarily high [24]. While this pathway is surely the primary source of NADPH in the endosymbionts, a significant quantity of NADPH may be produced also by an enzyme, GapN, [65] found in all Rhapolodian endosymbionts but rarely in free-living cyanobacteria. The enzyme is similar in critical sequence characteristics to a version of glyceraldehyde 3-phosphate dehydrogenase (GAPDH) that is unidirectional and generates NADPH but no ATP in the reaction [74, 75]. UCYN-A does not encode this enzyme. In wheat, a similar enzyme in conjunction with the conventional GAPDH has been associated with the coordination of NADPH and ATP production [76].

By fixing nitrogen at the same time their hosts are producing O_2_ through photosynthesis, the Rhopalodian endosymbionts face challenges greater than those faced by their closest free-living relatives. Perhaps for this reason, they possess an alternative respiratory terminal oxidase (ARTO), type 1, in addition to the conventional cytochrome oxidase [77] (Supplemental Table S2D). In the heterocyst-forming cyanobacterium, *Anabaena* PCC 7120, ARTO is expressed only in heterocysts and is required for nitrogenase activity [78]. The endosymbionts may also use ARTO to protect nitrogenase from O_2_, supplementing the effect of the unidirectional, O_2_-consuming uptake hydrogenase [79], encoded by the genomes of most cyanobacterial N_2_-fixers [80] and by all of the endosymbionts. All the endosymbionts lack flavodiiron proteins, e.g. Flv3, and so oxygen protection is unlikely through a Mehler-like reaction (reduction of O_2_ to H_2_O) [57, 81]. A true Mehler reactions (the reduction of O_2_ to H_2_O_2_) probably does not occur in any cyanobacterium [80] or endosymbiont.

Even though all the Rhopalodian endosymbionts lack PSI and PSII proteins, three of them (RgSB, EcSB, and EpSB) nonetheless encode all but three enzymes involved in chlorophyll biosynthesis [82, 83] (Supplemental Table S2D; see also [36]). Why has the partial pathway persisted? The first missing enzyme in the pathway is Lpor, light-dependent protochlorophyllide reductase, conserved in the set of core proteins. However that enzyme can be bypassed by the presence of ChlLNB, light-independent protochlorophyllide reductase [84], present in RgSB, EcSB, and EpSB. The endosymbionts also lack BciB, a reductase dispensible in low light [85]. Therefore, the absence of these two enzymes is probably not consequential, so long as a high rate of biosynthesis is not necessary.

However, RgSB, EcSB, and EpSB almost surely do not make chlorophyll. For one thing, EcSB at least does not have large amounts of chlorophyll as judged by fluorescence [24]. More importantly, the three endosymbionts lack the final enzyme in the pathway, geranylgeranyl diphosphate reductase (ChlP), containing pseudogenes instead. This enzyme converts the geranylgeranyl moiety within chlorophyllide *a* to the phytyl tail of chlorophyll *a*. Without this enzyme, *Synechocystis* PCC 6803 cannot grow photoautotrophically under any light regime [86], indicating that chlorophyllide *a* cannot substitute for chlorophyll *a* in photosynthesis.

Why maintain the seemingly defective pathway? The answer may lie in a proposal by Vavilin and Vermaas that free chlorophyll and precursors not bound to photosynthetic protein complexes may regulate one or more steps in the biosynthesis of tetrapyrroles [81]. Tetrapyrroles is a class that includes heme, chlorophyll, and pseudocobalamin [82]. *Synechocystis* has a set of Small Chlorophyll-*a*-binding Proteins (Scp) with a conserved chlorophyll-binding region at their C-termini. Ferrochetalase (HemH), at the crossroads between heme and chlorophyll biosynthetic pathways, has a similar C-terminus [87]. The binding of chlorophyll or a precursor to the binding-region was postulated to regulate the transcription or translation of upstream enzymes [81], and perhaps this is true also in RgSB, EcSB, and SpSB.

The presence or absence in endosymbionts of Scps or extended HemH correspond roughly to their chlorophyll biosynthetic capacities (Supplemental Fig. S09). Of the three Rhopalodian endosymbionts with a nearly complete chlorophyll biosynthesis pathway, one (EpSB) encodes HemH with a conserved C-terminal extension, One (EcSB) has an ortholog of ScpB from *Synechocystis*, with its conserved C-terminus. One (RgSB) encodes HemH with a C-terminal extension, although that terminus is dissimilar to C-termini from *Synechocystis*. Of the three endosymbionts with little or no chlorophyll-biosynthetic pathway, two (EaSB and EtSB) lack any Scp, and their HemH proteins have no C-terminal extension. The third, RaSB also lacks an extension on its HemH protein, but it has an ortholog of ScpE whose C-terminus shows six of the eight conserved residues. RaSB also has orthologs of 3 of 12 chlorophyll biosynthetic enzymes, so its pathway may be in an intermediate state of degradation. In summary, it is plausible that chlorophyllide *a* serves a regulatory role in some endosymbionts, not as a precursor to chlorophylls.

### Metabolic analysis – Central metabolites and cofactors

The greatest distinction between the capabilities of the Rhopalodian endosymbionts and UCYN-A as implied by their genomes is in the biosynthesis of central metabolites and cofactors. The genomes of the Rhopalodian endosymbionts imply complete biosynthetic pathways for purines, pyrimidines, fatty acids, phospholipids, and all 20 canonical amino acids (Fig. 4 and Supplemental Tables S2D, S2E, and S2F; see also Ref [74]).

The situation is quite different with UCYN-A, which lacks in its genome genes encoding all the enzymes of de novo biosynthesis of purines and most amino acids, to name only a few deficiencies. In some cases, metabolic holes are known to have been plugged by importing key enzymes from the algal host. Examples include threonine synthase and phosphoserine phosphatase, to complete the synthesis of threonine and serine, respectively, and orotate phosphoribosyltransferase to complete nucleotide biosynthesis [17]. In other cases, it must rely on the import of metabolites, e.g. through the purine salvage pathways, which remain intact. Since the UCYN-A genome implies no capacity to make the tricarboxylic acid cycle enzymes leading to 2-oxoglutarate (required for glutamate production), and since there’s no evidence of import of the enzymes [17], the endosymbiont must take in exogenous 2-oxoglutarate or glutamate. It must also procure a great many other metabolites [88], well beyond the range of the transporters encoded by its genome.

There is a similar distinction between the Rhopalodian endosymbionts and UCYN-A with respect to cofactor biosynthesis (Fig. 4 and Supplemental Table S2G and Figs S5-S8; see also Ref [74]). However, analysis is complicated by the imperfect knowledge of cyanobacterial cofactor biosynthetic pathways in general. At times, the most that can be said is that an endosymbiont’s deduced pathway is no more incomplete than that of free-living cyanobacteria, who surely make the cofactor. In this light, the Rhopalodian endosymbionts have as complete pathways as can be expected for the biosynthesis of six of the nine cofactors considered. In contrast, UCYN-A has only two cofactor pathways reasonably complete.

Of the three incomplete pathways, one, pantothenate/Coenzyme A biosynthesis [89, 90], may not deserve to be on the list (Supplemental Table S2G and Figs S7). Only one enzyme is missing in the Rhopalodian endosymbionts – aspartate 1-decarboxylase (PanD), leading to the production of β-alanine. That enzyme is also missing from all pico-cyanobacteria and 24% of other cyanobacteria. It’s a safe bet that all of them make Coenzyme A, so they probably have adopted a different route to produce β-alanine [89] or may take up the small amount of β-alanine needed for pantothenate biosynthesis from their hosts, as is seen in some insect-bacterial symbioses [91]. Whatever solution they found may also be available to the Rhopalodian endosymbionts, perhaps importing it through the N-II transporter [92] found in all endosymbionts (Supplemental Table S4B). EpSB, however, is defective in a second enzyme in the pathway, PanB, a core enzyme encoded by the other endosymbionts.

A second incomplete pathway, biotin biosynthesis [93], is more troublesome (Supplemental Table S2G and Fig. S05). The pathway may be divided into two stages: (a) the production of pimeloate esterified to either acyl-carrier protein or coenzyme A, and (b) the formation of the two rings of biotin. The first stage is catalyzed by diverse sets of enzymes. Neither the pathway used by *Escherichia coli* nor the different pathways used by *Bacillus subtilis* use enzymes with orthologs in any cyanobacterium, and it is unknown how any cyanobacterium accomplishes the first stage of biotin biosynthesis. The second stage, in contrast, is highly conserved amongst bacteria [91] and cyanobacteria. Orthologs of BioF, either BioU or BioA, BioD, BioB, and BirA are found in all or nearly all cyanobacteria, completing the pathway to protein-bound biotin. The Rhopalodian endosymbionts have these proteins as well, except (1) BioF is absent in all the endosymbionts, and (2) two related endosymbionts (EaSB and EtSB) lack BioU and BioB. While the biotin biosynthesis pathway is likely to be non-functional in these two endosymbionts, it is possible that the pathway in the other four retain function, if they are able to import the product of BioF (7-amino-8-oxononanoate) from their hosts. The endosymbionts may also import host-synthesized biotin through a protein found in all of them (as well as in UCYN-A) that is orthologous to a proven biotin transporter from *Rhodobacter capsulatus* [94] (also orthologous to *Synechocystis* protein Slr1365).

The third incomplete cofactor pathway, folate biosynthesis [95], poses problems at one entry point and at its final step (Table S12, Fig. S06). The synthesis of folate requires the production of p-aminobenzoate (PABA). This is conventionally achieved through three reactions starting from chorismite, catalyzed by PabAabc (also called PabA/PabB and PabC). All the Rhopalodian endosymbionts lack orthologs for the first protein, PabAa, even though orthologs are found in the core set. If the endosymbionts are able somehow to acquire PABA, then the remainder of the pathway is fine until the step leading to the production of tetrahydrofolate, the cofactor required for several enzymatic reactions. The enzyme used by *E. coli* for this final step, dihydrofolate reductase (FolA; b0048), and by most bacteria [93], is similar to proteins found in only 15% of cyanobacteria, none closely related to the endosymbionts. It is not clear how most cyanobacteria catalyze the final reduction to tetrahydrofolate, but many alternatives are possible [93]. As a fallback position, the endosymbionts may be able to take up folate or 5-formyl-tetrahydrofolate from their hosts, using transporters orthologous to a *Synechocystis* protein (Slr0642) with proven ability to transport both [96]. The protein is found in all Rhopalodian endosymbionts but not in UCYN-A.

The role and synthesis of pseudocobalamin warrants special attention, because there is reason to believe that some of the endosymbionts provide their hosts with this cofactor in addition to fixed nitrogen. Cobamides are required cofactors in several reactions and are synthesized only by bacteria [82]. In most cases, the biologically relevant cobamide is cobalamin (Vitamin B_12_). However, almost all cyanobacteria encode enzymes that lead not to cobalamin but to pseudocobalamin instead, [97] which differs from cobalamin in the presence of adenine in place of 2,6-dimethylbenzamidazol (DMB) as a ligand.

While land plants generally rely on cobalamin-independent enzymes, animals and some algae must take up exogenous cobalamin for survival [98]. This is true for 62% of diatoms and 64% of haptophytes [96]. Some can also grow when provided with exogenous pseudocobalamin but only when supplemented with DMB [95]. Coale et al. (2024) [17] showed that *B. bigelowii*, the host of UCYN-A, expresses two nuclear-encoded transcripts related to cobalamin biosynthesis (Supplemental Fig. S08), even though the alga encodes no other enzymes of cobamide biosynthesis:

- KC1-P2-N_k31_Locus_18619_Transcript_1_1: Encodes BluB (5,6-dimethylbenzimidazole synthase, which makes DMB
- KC1-P2_N3_k31_Locus_9846_Transcript_1_1: Encodes conventional CobT (DMB phosphoribosyltransferase), which prepares DMB for attachment to the cobamide.

This raises the possibility that the host is able to remodel the pseudocobalamin (or an intermediate) produced by UCYN-A to meet its need for cobalamin. Conceivably, the Rhopalodian hosts have discovered the same trick.

Like UCYN-A, four Rhopalodian endosymbionts (RgSB, RaSB, EcSB, and EpSB) encode complete biosynthetic pathways to produce pseudocobalamin, to the extent that the steps are currently understood (Supplemental Table S2G and Fig. S08). Genes encoding the enzymes for two steps (SirC and CobC) have not yet been found in any cyanobacterial or endosymbiont genome. CobC encodes a phosphatase that catalyzes the final step in cobalamin and pseudocobalamin biosynthesis. There is confusion in the literature concerning the other missing enzyme, SirC, encoding precorrin-2 dehydrogenase. In *E. coli*, its function is handled by CysG, an enzyme with three distinct regions, each catalyzing a different reaction [99]. These regions correspond to three separate enzymes in *Bacillus subtilis* (SirA, SirC, and SirB), catalyzing reactions leading to seroheme [100]. The first two are also essential for the synthesis of cobalamin (SirA is synonymous with CobA) [82]. While CysG bears a high degree of similarity to cyanobacterial enzymes, the similarity lies only in two of the three regions, not the region corresponding to SirC. Since many cyanobacteria with these uncertainties demonstrably produce pseudocobalamin [95], it is likely that this is true as well of RgSB, RaSB, EcSB, EpSB, and UCYN-A.

It remains a mystery how EaSB and EtSB manage to survive, unable to synthesize pseudocobalamin but still evidently relying on two pseudocobalamin-dependent enzymes: methionine synthase (MetH) and epoxyqueuosine reductase (QueG; used to modify tRNA) [101]. Both are present in all Rhopalodian endosymbionts. However, unlike many bacteria [99] and most cyanobacteria, the endosymbionts and their close relatives use the cobamide-independent form of ribonucleotide reductase. Perhaps EaSB and EtSB can scrounge enough pseudocobalamin from the aqueous environment [95, 102] as does the pseudocobalamin auxotroph *Synechococcus* PCC 7002 [103]. It is less likely that the two endosymbionts make use of cobalamin in place of pseudocobalamin. Cyanobacterial MetH binds cobalamin very poorly [104], and mutation would need to have taken place to enable the two endosymbionts to share the cobalamin used by its diatom host.

### Carbon transport

**Fig. 6:**
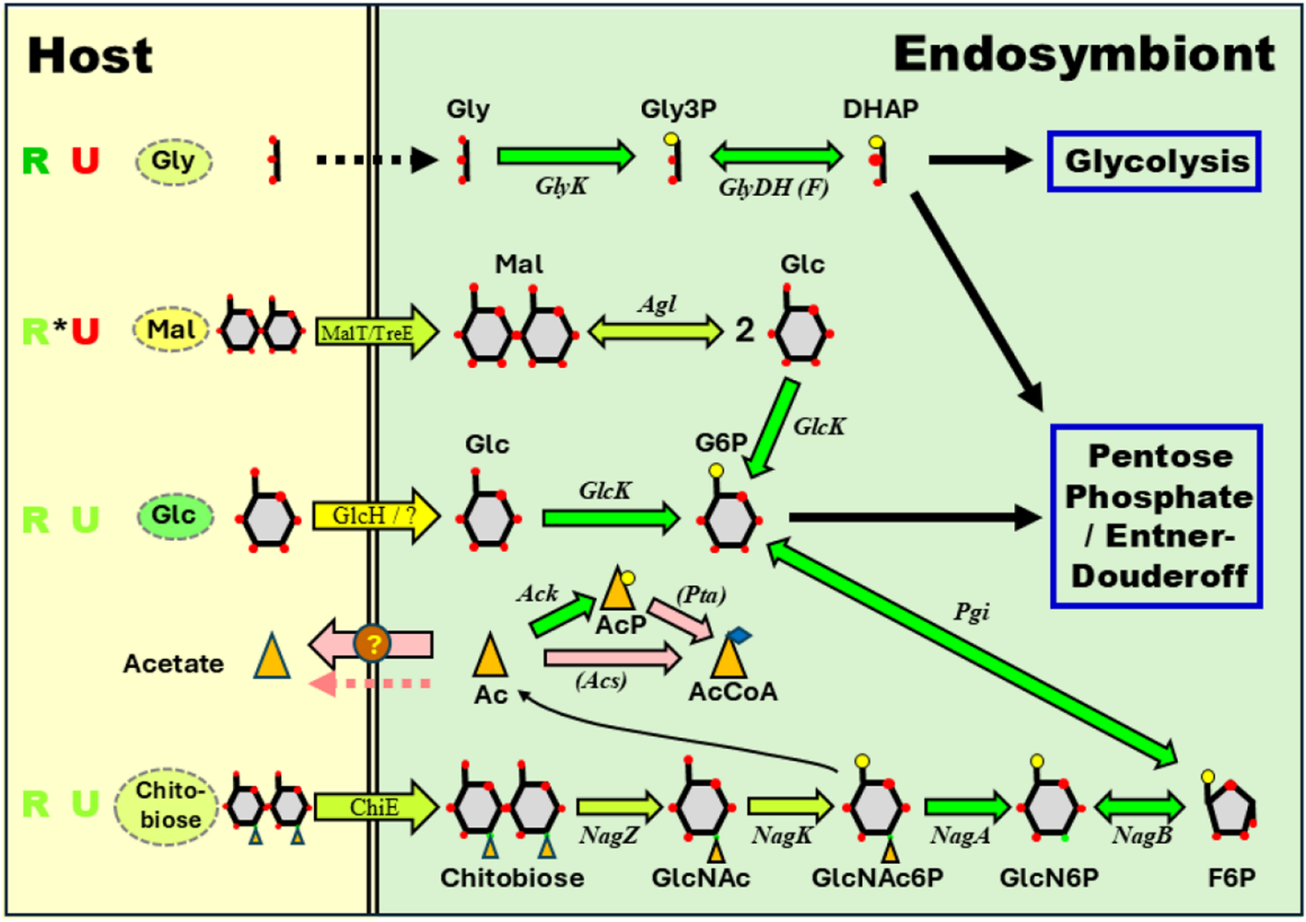
Candidate pathways for uptake of carbon from host to endosymbiont. See Supplemental Table S4A for list of enzymes, transporters, their abbreviations, and associated genes in the different endosymbionts. Arrows across the double line indicate transporters at a membrane interface (double line) between an endosymbiont and the host. A dashed arrow indicates diffusion of the compound. The colors of the arrows indicate the perceived likelihood that the indicated reaction takes place in Rhopalodian endosymbionts: Green (very likely), yellow-green (possibly), yellow (hypothetical), pink (no evidence, where evidence would be expected. The colors of the ovals in the host compartment indicate in the same way the perceived likelihood that the compound is present in diatom hosts. The colors of “R” and “U” indicate the overall perceived likelihood that the route is present in Rhopalodian endosymbionts or UCYN-A, respectively. An asterisk indicates that the route is surely not present in certain endosymbionts (see Supplemental Table S4A). Abbreviations: Gly, glycerol; Gly3P, glycerol-3-phosphate; DHAP, dihydroxyacetone phosphate; Mal, maltose; Glc, glucose; G6P, glucose-6-phosphate; Ac, acetate; AcP, acetate phosphate; AcCoA, acetyl Coenzyme A; GlcNAc, N-acetylglucosamine; GlcNAc6P, N-acetylglucosamine-6-phosphate; GlcN6P, glucosamine-6-phosphate; F6P, fructose-6-phosphate

One might take away from the previous sections that the Rhopalodian endosymbionts are independent of their hosts with respect to major metabolic and informational categories. However, they are assuredly dependent in one important area. By having lost photosystems I and II and various proteins important in carbon fixation, they necessarily rely on their hosts for a source of carbon, both to synthesize intermediary metabolites and to power metabolism. It is important to determine to the extent possible what that carbon source is. The discussion below is summarized in Fig. 6 and Supplemental Table S4A, and a more detailed discussion of this question is available elsewhere regarding RgSB and EtSB [74].

#### Glycerol and other simple sugars

Perhaps the first candidate for a carbon source is glycerol. Free-living Cro51142 can be trained to grow well in the dark on 50 mM glycerol. Glucose does not support growth, and its ability to enhance growth in the light is small or non-existent [105, 106]. All the Rhopalodian endosymbionts (but not UCYN-A) have the two enzymes necessary to take glycerol to the glycolytic intermediate dihydroxy acetone phosphate: glycerol kinase and FAD-dependent glycerol-3-phosphate dehydrogenase (Fig. 6 and Supplemental Table S4A). In fact, the endosymbionts are better placed than most free-living strains, such as S8802, which have only NAD(P)-dependent glycerol-3-phosphate dehydrogenase. That form energetically favors the *production* of glycerol-3-phosphate rather than its *consumption*.

What remains to be established is whether the diatom hosts produce glycerol at a level high enough to enable sufficient uptake by the endosymbionts. Glycerol can enter cells by diffusion, facilitated transport, or active transport [107]. In *E. coli*, a glycerol concentration above 1 mM is sufficient to promote growth, unaffected by the presence or absence of a glycerol facilitator protein [108], and so if the host concentration of glycerol exceeds this amount, then a transporter may not be necessary. Is this likely? Glycerol is used as an osmolyte in some halophilic algae [109] and certain halotolerant diatoms [110, 111]. One of the latter increased its internal concentration of glycerol from 5 mM during growth in 150 mM salt to 70 mM during growth in 700 mM salt (a bit higher than the level in seawater) [108]. However, one might not expect freshwater diatoms (e.g., the hosts of five of the six endosymbionts considered here) to maintain an appreciable level of glycerol.

Perhaps glycerol is the answer to the carbon-source puzzle, but if not, then sugars come to mind as obvious candidates. This is because glucose, fructose, and sucrose are preferred carbon sources by cyanobacteria that are good heterotrophs [112, 113]. One of them, *Nostoc punctiforme*, requires the glucose transporter GlcP for the establishment of symbiosis with the hornwort *Anthoceros punctatus* [114]. Unfortunately, good matches to the Nostoc protein is found in none of the closest relatives of the endosymbionts, and no ortholog is present in any Rhopalodian endosymbiont nor in UCYN-A. The endosymbionts do possess orthologs of GlcH, a protein from the marine picocyanobacterium *Prochlorococcus* ss120 that supports glucose uptake [115]. However, it appears not to have that function in cyanobacteria more closely related to the endosymbionts [74]. Other routes by which glucose, fructose, or sucrose might serve as a carbon source are unlikely to be pertinent in the endosymbionts [74].

#### Maltose

Three other sugars warrant consideration as possible carbon sources. Maltose (α(1 → 4) glucose-glucose disaccharide) is an intriguing possibility (Fig. 6 and Supplemental Table S4A), because four endosymbionts carry proteins orthologous to a proven maltose transporters (MalT) from three diverse heterotrophic bacteria [116–118]. These three members of the major facilitator superfamily (MFS) share 126 positions of identity, of which 71%−72% are shared as well with the endosymbiont orthologs (Supplemental Fig. S10). In contrast, other members of the MFS family that transport lactose or galactose match only 18% of these conserved positions, so the endosymbiont proteins are similar only to MFS proteins that transport maltose.

If we accept that some endosymbionts can take up maltose (not EaSB or EtSB), then they should have a means of metabolizing the sugar. Two routes are known: maltose phosphorylase, which produces glucose-1-phosphate plus unphosphorylated glucose; and α-glucosidase, which (if it acts on maltose) produces two molecules of glucose. No endosymbiont or close relative contains anything similar to the proven maltose phosphorylase from *Bacillus subtilis* [119], but all Rhopalodian endosymbionts encode two proteins (named here aGal1 and aGal2) that may substitute, generally annotated as α-glucosidase (Supplemental Table S4A). It remains to be determined experimentally whether either one acts on α(1 → 4) linkages (such as those in maltose or instead on α(1 → 6) linkages (such as those found in branched polysaccharides). However there is good reason to believe this is the case. The sequences of enzymes with known α(1 → 4) and α(1 → 6) activities [120–123] were collected, aligned, and used as the basis of a phylogenetic tree (Supplemental Fig. S11). It is apparent that the aGal1 α-glucosidases cluster with proven α(1 → 4) glucosidases, some of which are known to act on maltose, while the aGal2 α-glucosidases cluster with a mixed collection of enzymes. However, Okuyama et al. (2016) found that in their set of enzymes, the linkage specificity of the enzymes could be accurately predicted by the amino acid next to a universally conserved catalytic aspartate residue, alanine or threonine predicting α(1 → 4) linkage specificity and valine predicting α(1 → 6) linkage specificity [120]. Both aGal1 and aGal2 have alanine in the critical position, so possibly both enzymes act on α(1 → 4) linkages.

The endosymbionts can take up and metabolize maltose only if it is present outside their membranes. Here there is a problem. Maltose is common in land plants, which use starch as a storage form. But diatoms instead store glucose as chrysolaminarin [124], a mixed β−1,3- and β**-**1–6-glucan. Breakdown of the polymer should produce not maltose but glucose and β-linked oligosaccharides [122]. Maltose would therefore not be expected to be available to the endosymbionts.

#### Amino sugars chitobiose and N-acetylneuraminic acid

The two remaining candidates to discuss are both amino sugars: the disaccharide chitobiose, consisting of two molecules of N-acetylglucosamine (GlcNAc) in a β(1→4) linkage, and the simple sugar N-acetylneuraminic acid (NeuN5Ac) (Fig. 6 and Supplemental Table S4A). In both cases, the same questions arise: (1) Can the compound be transported into the endosymbiont? (2) Can the endosymbiont metabolize it? and (3) Is the host likely to provide the compound?

In the case of chitobiose, the answers to all three questions would seem to be the same: “probably”. Some diatoms (including one of the same order as the Rhopalodia) have been found capable of synthesizing chitin, a β(1→4)-linked polymer of GlcNAc [125], and one might expect to find its breakdown products, including chitobiose, in such diatoms. If the endosymbionts hosts are amongst this group, then it is very likely that the endosymbionts will be able to take up the compound. This is because GlcNAc along with N-acetylmuramic acid form the backbone of peptidoglycan in the cell walls of bacteria, including cyanobacteria [126, 127]. Peptidoglycan has been observed in UCYN-A [128] and surrounds probably all the endosymbionts. The recycling of peptidoglycan components is common amongst all bacteria [129] and involves the uptake of short GlcNAc-containing disaccharides and other compounds from outside the cell [130].

All of the endosymbionts (including UCYN-A) and almost all cyanobacteria appear to possess the enzymes required to convert chitobiose into glycolytic intermediates. The process begins with a β-N-acetylglucosaminidase (NagZ), which can release GlcNAc from a peptidoglycan fragment [131, 132]. An enzyme usually annotated ambiguously as “β-glucosidase”, exhibits in all endosymbionts and almost all cyanobacteria an amino acid motif diagnostic of β-N-acetylglucosaminidases [133]. In other organisms, some, but not all of these enzymes are able to act on chitobiose (GlcNAc-GlcNAc) [129, 134, 135]. In the endosymbionts, NagZ lacks a detectable transit peptide, so it would be predicted to act within the cell, like NagZ from Gram-negative bacteria and unlike those from Gram-positive bacteria [136]. The remaining steps are catalyzed by GlnNAc kinase (NagK), GlcNAc-6-phosphate deacetylase (NagA), and GlcN-6-phosphate deamidase (NagB). The latter two appear to be present in all Rhopalodian endosymbionts, while NagK is probably accounted for by an ROK-family enzyme [74].

The endosymbionts are certainly able to metabolize exogenous peptidoglycan-derived GlcNAC-disaccharides, and should therefore be able to take them up – perhaps chitobiose as well. This may be achieved through a transporter found in all endosymbionts and most cyanobacteria (Supplemental Table S4A). The most similar transporter in the Transporter Classification Database (TCDB) [137] is ChiE from *Thermotoga maritima* [138]. This was judged to be a chitobiose transporter, because: (a) *T. maritima* is able to grow on chitobiose, (b) it is regulated by a protein that binds chitobiose, and (c) It is encoded by a gene within an operon that also contains genes encoding NagZ, NagA, and NagB, suggesting a functional relationship with the enzymes that metabolize chitobiose.

There is also circumstantial evidence connecting ChiE to chitobiose in the endosymbionts. ChiE is the substrate-binding component of an ABC transporter [139, 140]. It lies adjacent in the genome of *T. maritima* to two genes encoding permeases, NagF and NagG, presumably participating in the Chi ABC transporter. Orthologs to the two permeases are found in all endosymbionts. In UCYN-A, one lies nearby the genes encoding NagK and NagZ. All in all, NagF and NagG probably combine with ChiE to form an intact transporter. While chitobiose is not a breakdown product of peptidoglycan, the transporter that brings in GlcNAc-disaccharides may have been repurposed to transport chitobiose as a high volume carbon source for the endosymbionts.

The appeal of the second amino sugar candidate, NeuNAc, lies primarily in the fact that all Rhopalodian endosymbionts have orthologs of NanT, the putative transporter of NeuNAc, even though orthologs are absent in the endosymbionts’ closest cyanobacterial relatives and are rare in cyanobacteria in general. However, the presence of NeuNAc has not been described in diatoms, and the metabolism of NeuNAc within the endosymbionts poses certain problems, discussed previously [74].

### Nitrogen transport

N_2_-fixing cyanobacteria release very little fixed nitrogen into their environments [141], but substantial release is exactly what diatom hosts are asking of their endosymbionts. We have two models of the export of fixed nitrogen by free-living cyanobacteria. One is the export of primarily glutamine and aspartate-arginine from heterocysts to adjacent vegetative cells [142, 143]. The currently prevailing view is that this export takes place through physical junctions between adjacent cells [144], requiring several proteins, e.g. SepJ, FraC, and FraD [140]. Orthologs to these proteins are not found in unicellular cyanobacteria nor in the endosymbionts.

A more useful model may be the export of nitrogen from symbiotic heterocyst-forming cyanobacteria to their hosts, within extracellular associations (with cycads, bryophytes, ferns, and diatoms) [139, 145] and intracellular associations (with the angiosperm Gunnera [146] and the diatom Hemiaulus hauckii [143]). In these cases (with the possible exception of the Nostoc-cycad symbiosis [147]), the host gains nitrogen in the form of NH_4_^+^, made possible by the low activity of glutamine synthetase [148]. Glutamine synthetase catalyzes the first reaction in cyanobacteria to incorporate fixed ammonia into an organic molecule [149], and low activity, either by direct regulatory control or restriction on the synthesis of the enzyme, should leave an endosymbiont awash in NH_4_^+^ during a period of nitrogen fixation.

**Fig. 7:**
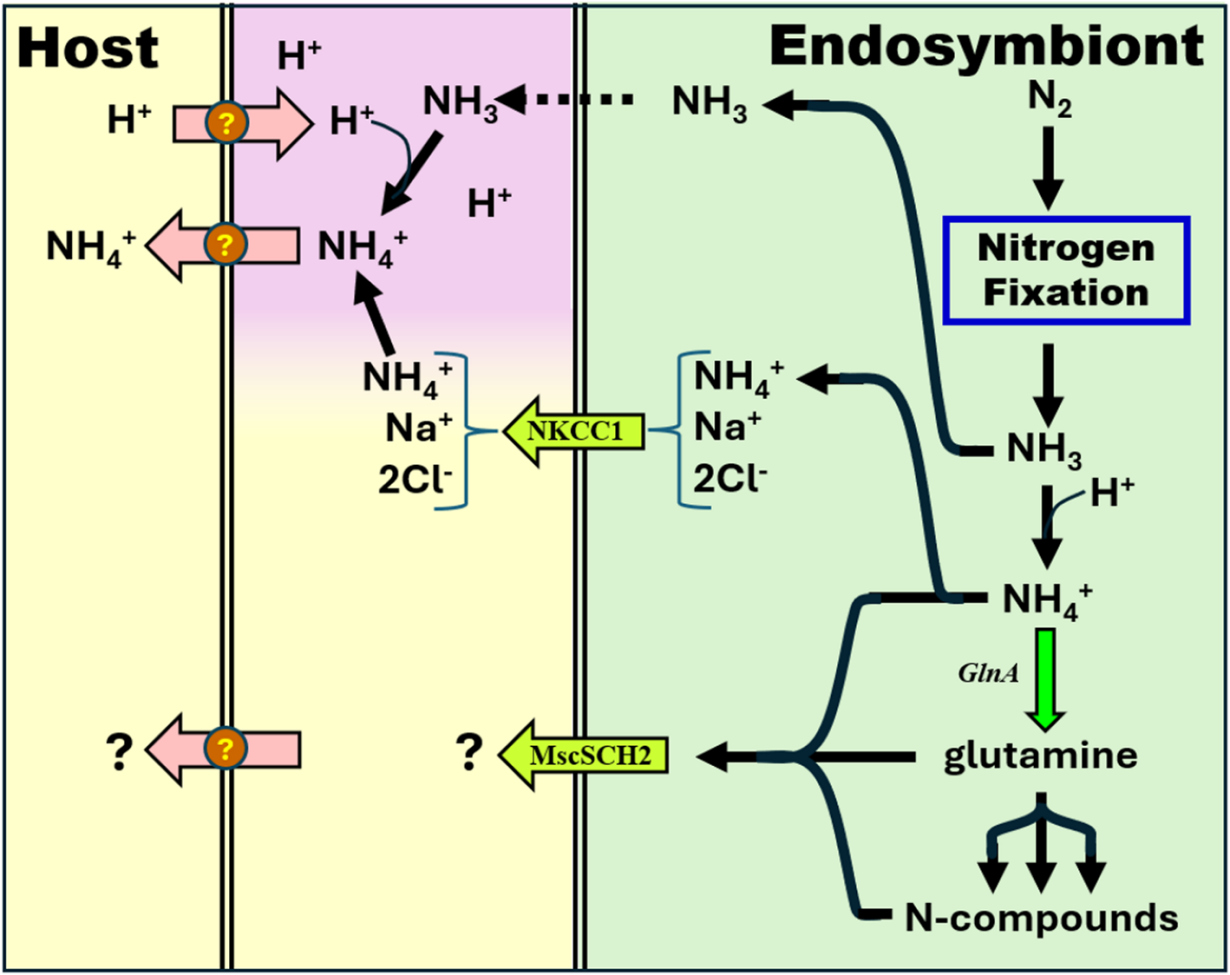
Candidate pathways for efflux of nitrogen from endosymbiont to host. See Supplemental Table S4A for list of transporters, their abbreviations, and associated genes in the different endosymbionts. Color conventions are the same as in Fig. 6. The host cytoplasm is shown separated from the endosymbiont a symbiotic cavity bounded by a host-derived membrane (left double line) and endosymbiont membrane. In one scenario, the symbiotic cavity is postulated to be acidified (pink) as described in the text

A high concentration of NH_4_^+^ within an endosymbiont does not by itself do the host any good. A large amount of the fixed nitrogen must somehow find its way into the host’s cytoplasm. A process by which this may happen has been proposed to explain the transfer of fixed nitrogen from rhizobial bacteroids to their legume hosts (Fig. 7, top) [150, 151]:

- The NH_3_ fixed within bacteroids diffuses across the bacteroid membrane [152]. At physiological pH, it is in equilibrium with NH_4_^+^.
- The ammonia is trapped as NH_4_^+^ outside the bacteroids in a symbiotic cavity, because the host has pumped acid into the cavity surrounding the bacteroid to lower the pH.
- The host is protected from the low pH by a symbiotic membrane, separating the symbiotic cavity from the cytoplasm.
- The host takes up NH_4_^+^ using a host-encoded active transporter on the symbiotic membrane.
- Import of NH_4_^+^ by the bacteroid is repressed, to avoid reuptake.

A critical requirement for this scheme is the presence of a symbiotic space. Such spaces provided by the host (either extracellularly or intracellularly) are known to enclose all characterized cyanobacterial symbioses [153], with the exception of Richelia/diatom associations [154]. A Rhopalodian endosymbiont whose genome has not been sequenced also has been shown to be enclosed in a host-derived membrane [155], as has UCYN-A [126, 156]. Insect nutritional endosymbionts are also commonly encased in host-derived membranes [91]. If the diatom host directs an H^+^-transporting ATPase and an NH_4_^+^ transporter to the symbiotic membrane, there’s little left for the endosymbiont to do but shut down its own Amt1 NH_4_^+^ transporter (Supplemental Table S4B) and fix N_2_. UCYN-A lacks any NH_4_^+^ transporter, so that step is unnecessary.

There are alternative routes the endosymbiont might employ to feed NH_4_^+^ to its host. In a careful study of the transporters encoded by heterocyst-forming cyanobacterial strains in association with diatoms, Nieves-Morión et al. identified a remarkable transporter that might fill the bill [143]. The human protein NKCC1 [157] in the Cation-Chloride-Cotransporter (CCC) family is highly similar to a protein in each of the Richelia strains they considered [143] and is also present in all Rhopalodian endosymbionts as well as in UCYN-A (Fig. 7, Supplemental Table S4B). The typical role of the protein in humans is to cotransport Na^+^, K^+^, and Cl^−^ in either direction (regardless of the orientation of the transporter), governed by the [Cl^−^] gradient [158], but its most notable feature for our purposes is that it is also able to transport NH_4_^+^ (replacing K^+^) [159].

NKCC1 consists of three regions [160]: an N-terminal soluble region associated with the site of regulation by phosphorylation [155], a central region with twelve transmembrane domains, and a C-terminal soluble region that may be related to dimer stabilization and that interacts with transmembrane domains [158]. All eubacterial matches to NKCC1 are limited to the central and C-terminal regions, constituting 76% of its length and missing the 24% apparently devoted to regulation. Proteins very similar to NKCC1 are found in many cyanobacteria but rarely in heterotrophic bacteria.

A third possibility is that NH_4_^+^ or another nitrogenous compound might exit endosymbionts through relatively non-specific transporters of the mechanosensitive channel family (MscS), which function to release solutes in response to hypotonic conditions [161]. Perhaps the best studied is MscSCG and MscSCG2 from *Corynebacterium glutamicum* [162, 163], which has been exploited for the industrial production of glutamate [164]. These transporters are also able to facilitate the export of other amino acids [159, 165]. MscS transporters are found in all cyanobacteria, almost always in multiple versions. All the endosymbionts have orthologs of MscSCG2 (Supplemental Table S4B). Mutations in E. coli that render an MscS-family protein constitutively expressed are lethal [166], but *C. glutamicum* is viable with constitutive expression of either MscSCG or MscSCG2, [161, 167, 168] so it is conceivable that the natural orthologs of the protein in endosymbionts might have been repurposed to mediate the routine export of a nitrogenous compound.

Other less likely candidates in the Dicarboxylate/Amino Acid: Cation Symporter (DAACS) and Drug/Metabolite Transport (DMT) families have been discussed elsewhere [74, 143].

## Discussion

What is required from an existing nitrogen-fixing endosymbiont that would allow it to interact productively with a new host, specifically, with a crop plant? In this work, I have examined three parts of that question: (1) Do the genomes of the Rhopalodian endosymbionts encode the proteins required to meet its metabolic needs? (2) What carbon source from the host is required by the endosymbionts? and (3) By what process is fixed nitrogen exported to the hosts of the endosymbionts?

### Metabolic independence of endosymbionts

There is prior reason to expect that the Rhopalodian endosymbionts might live primarily within the limits of their own resources. A proteomic study of the EcSB/*Epithemia clementina* association identified only six host-encoded proteins in the isolated endosymbiont, none of any obvious importance [169]. This is in stark contrast to the hundreds of proteins imported by UCYN-A [17] and by the *Paulinella* chromatophore [170]. However, even one imported protein would be of clear significance, if it were shown that it had indeed traversed the membranes separating the host cytoplasm from the endosymbiont, since that would signify that a mechanism to import proteins had been established. This remains to be demonstrated.

Given the apparent absence of host contributions to the proteome of Rhopalodian endosymbionts, perhaps it is no surprise that the endosymbionts appear to encode all the proteins it needs for the synthesis of macromolecules and their components, for ATP and NADPH production, and the cofactors needed for metabolic processes (with some reservations — see below). This conclusion must be tempered by humility: there surely are proteins important for survival that I did not consider, proteins whose functions may conceivably be provided by the host. They presumably would be contained within the list of 512 core proteins absent in endosymbiont genomes (Table 1, Supplemental Table S3B). These lists contain many proteins that one would expect to be absent in the endosymbionts (e.g. PSII proteins), and nothing in them stands out to me as a protein that would be needed for endosymbiont function.

Cofactors require special consideration regarding the ability of a Rhopalodian endosymbiont to function in a new host. At one extreme, there is pseudocobalamin. Since land plants do not make or utilize cobamides [171], it is essential that the endosymbiont be able to synthesize its own (or, alternatively, be engineered to use cobamide-independent enzymes to replace the few that require the cofactor). In the case of some of the endosymbionts (RgSB, RaSB, EcSB, and EpSB), this condition appears to be met.

Their biotin biosynthesis pathways of the endosymbionts are incomplete, however. Land plants may be able to accommodate their needs, since the biotin biosynthetic pathway is split (at least in *Arabidopsis*), with the initial steps taking place in the cytoplasm and the final steps in the mitochondria [169, 172] The same four endosymbionts are deficient only in the first enzyme of the pathway, so they may be able to join mitochondria in taking up the intermediate substrate, 7,8-diaminonanoate. All endosymbionts also encode a transporter of biotin, which must exist in the plant cytosol.

Folate biosynthesis is deficient in the endosymbionts because they appear to lack the ability to make PABA, a starting point of the pathway, but this may not be a problem. In *Arabidopsis*, PABA is synthesized in chloroplasts, exported to the cytosol, and imported by mitochondria, where synthesis of tetrahydrofolate is completed [173]. Admittedly, it’s not clear how the endosymbionts could take up the cytosolic PABA. If, however, a mechanism exists, then that would explain why the endosymbionts have preserved the downstream enzymes of the pathway (except possibly the last). If it doesn’t, then it may be enough that all Rhopalodian endosymbionts have orthologs of a transporter capable of taking up 5-formyl-tetrahydrofolate from the plant host [94].

In short, the metabolism implied by the genomes of the endosymbionts may suffice to enable them to meet their metabolic needs within a new plant host.

### Import of a carbon source and export of fixed nitrogen

All that metabolic capability counts for nothing unless the endosymbionts can gain from their hosts a source of carbon, as starting points for metabolic reactions and as an energy rich compound to power the production of NADPH and ATP. Glycerol would be an obvious choice, as it is the only carbon source shown to support growth of Rip8802. Furthermore, the endosymbionts have all the enzymes (including an unusual glycerol-3-phosphate dehydrogenase) required to channel glycerol into central metabolism. However, while significant levels of glycerol are not uncommon in marine diatoms, they wouldn’t be expected in freshwater diatoms, such as those that host five of the six endosymbionts. Glycerol would be a stronger candidate if it were established that all the symbioses derived from a single acquisition of a cyanobacterium by a marine diatom (see below), one that retained production of glycerol even after its progeny colonized freshwater habitats. Of course, the candidacy of glycerol could be assessed by the simple act of measuring glycerol in the host diatom strains.

It may be that glycerol – agreeable to both the marine diatom and its newly acquired cyanobacterium – was just a temporary solution, replaced over the time required to adapt to freshwater by a different carbon source. I’ve suggested that chitobiose is a reasonable candidate, as it is found in some diatoms, and endosymbionts are likely to take it up and metabolize it to glycolytic intermediates. This would require ramping up a transporter designed to act on low levels of peptidoglycan breakdown products so that it can provide the high levels of carbon required by nitrogen-fixation. Again, this hypothesis could be tested by measuring chitobiose in the different strains.

Neither glycerol nor chitobiose is likely to be found in the cytosol of crop plants. It will probably be necessary to engineer an endosymbiont to use a glucose transporter. Or perhaps the mysterious endosymbiont protein similar to maltose transporters will serve.

The mechanism of nitrogen export from the endosymbiont to its host is also unclear. One possibility is that NH_3_ resulting from N_2_-fixation diffuses into an acidified space between the cyanobacteria-derived and host-derived membranes, analogous to the mechanism by which N-transfer is thought to take place in root nodules between bacteroids and legumes [148, 149]. However, this mechanism relies on the host to provide a membrane to surround the nitroplast and stock the membrane with enzymes to acidify the internal space and to take up NH_4_^+^. Crop plants are unlikely to be able to accommodate these needs, so a different transport mechanism would need to be engineered into the nitroplast.

Two possible mechanisms of nitrogen export suggested by the endosymbiont genomes would be more practical in crop plants. Transporters similar to NKCC1 from humans in all Rhopalodian endosymbionts could facilitate the export of fixed nitrogen in the form of NH_4_^+^ from the endosymbiont to the cytosol of the new host (presuming that there is no host-provided membrane surrounding the endosymbiont). Alternatively, endosymbiont orthologs to MscSCG2 from *C. glutamicum* might act as a conduit for NH_4_^+^; they could also serve to export an organic form of fixed nitrogen. The physiological response of plant cells to a large influx of fixed nitrogen (particularly NH_4_^+^ [174]) is a concern. A first step is determining the form of nitrogen that passes between endosymbionts and their natural hosts. The activity of glutamine synthetase in endosymbionts would be particularly interesting to know.

### Evolution of the Rhopalodian endosymbionts

The Rhopalodian diatoms are believed to have arisen from a common marine ancestor [41], from which the freshwater strains were derived. This suggests at least the following three scenarios to explain the presence of the endosymbionts:

1. *Multiple acquisitions (marine and freshwater)*: A marine Rhopalodian diatom acquired a cyanobacterium, leading to the endosymbiont EpSB, while one or more freshwater Rhopalodian diatoms separately acquired a related cyanobacterium, leading to the other five endosymbionts.
2. *Single Acquisition (marine)*: An ancestral marine Rhopalodian diatom acquired a cyanobacterium, and all endosymbionts are derived from this ancestor, some of whose progeny learned to live in freshwater.
3. *Single Acquisition (freshwater)*: A more recent freshwater Rhopalodian diatom acquired a cyanobacterium, and all endosymbionts are derived from this ancestor. An ancestor specifically of EpSB transitioned to a marine environment. While freshwater/marine transitions are not uncommon in the distantly related diatom order Thalassiosirales [175], it is not clear whether they take place in the Rhopalodiales [41].

The multiple acquisition hypothesis is made more plausible by the apparent age of EpSB, the endosymbiont of the lone marine diatom considered in this study. EpSB has a smaller genome than the other endosymbionts (Fig. 1), and preliminary evidence suggests that it has far fewer broken genes (see Supplemental Table S2A; filter each column for “(“, the symbol for broken genes), consistent with a gene density that is intermediate between those of other endosymbionts and the older UCYN-A and *Paulinella* chromatophore (Fig. 1). Perhaps these characteristics arise from greater evolutionary time to dispose of the genetic debris [176]. Alternatively, EpSB might simply be more efficient in garbage collection.

There are problems with the idea of multiple acquisition events, however. It implies that the hypothetical freshwater cyanobacterial ancestor of the five freshwater endosymbionts is closer phylogenetically to the hypothetical marine cyanobacterial ancestor of EpSB than to the existing freshwater *Rippkaea* PCC 8802 (Rip8802), the closest known free-living relative. This is possible, but supporting the idea of a single marine ancestor (Hypothesis #2) is the finding that proteins with orthologs in all the endosymbionts but not in Rip8802 have orthologs primarily in marine cyanobacteria (Table 2, Supplemental Table S3C). For example, while most cyanobacteria possess Fe-dependent superoxide dismutase (FeSOD), a minority has a Ni-dependent form of the enzyme (NiSOD), almost all marine strains (Table 2 and Ref [177]). All the endosymbionts have NiSOD. The strong association of NiSOD and marine habitats extend to heterotrophic bacteria as well, and it has been suggested that the preference is in response to the deficiency of iron in oceans [178]. The presence of NiSOD in the endosymbionts would make sense if there were a single progenitor, a free-living marine cyanobacterium.

## Conclusions

The goal of this work was to assess whether any dependency of endosymbionts on their Rhopalodian hosts would preclude the transfer of the endosymbiont to a different host. A close examination of the genomes of six endosymbionts of Rhopalodian diatoms shows no obvious metabolic dependency on their hosts, except for possibly some cofactors needed in very low amounts. This contrasts with the many dependencies evident from the genomes of two other cyanobacteria-derived endosymbionts: UCYN-A and the chromatophore from *Paulinella* chromatophore. Furthermore, the endosymbionts possess some unusual enzymatic capabilities that may enable them to manage the high need for reductant in the absence of PSII (GapN), withstand the production of O_2_ by host photosynthesis (ARTO), and make more efficient use of glycerol as a carbon source (FAD-dependent glycerol-3-phosphate dehydrogenase).

It would be a mistake, however, to jump from this finding to the conclusion that Rhopalodian endosymbionts are ready to thrive within crop plants. This is highly improbable. First, crop plants are unlikely to provide a carbon source the endosymbionts seem equipped to accept and the mechanism of nitrogen export by the endosymbionts may be incompatible with crop plant physiology. Experiments on a model Rhopalodian endosymbiont are necessary to clarify these issues. Second, there was nothing presented here regarding the integration of cell division and division of the endosymbionts, as has been demonstrated with UCYN-A [17]. The Rhopalodian endosymbionts evidently employ some way of ensuring defined partitioning into daughter cells [26, 179] (despite a report of loss of endosymbionts after extended growth in the laboratory [37]), but until the mechanism is known, it is impossible to say whether it could function in alternative hosts.

Clearly, a good deal of laboratory experimentation is required to form a basis for the transfer of an endosymbiont to a foreign host. Of the six endosymbionts considered here, EcSB may be the most appropriate as a model system. Unlike some of the others (EaSB, EtSB, and RaSB), it appears able to synthesize pseudocobalamin. Unlike EpSB, it has what may be a complete pathway to synthesize pantothenate and more complete pathway for folate. Unlike RgSB, it has been grown in the laboratory, at least as a unialgal culture [24]. But there is no need to create all the tools required to tame a new model organism. A good path forward might be to couple basic experiments in EcSB with a study of the EcSB endosymbiont transferred to a related diatom, *Phaeodactylum tricornutum*, that offers a wealth of laboratory capabilities [180]. This prospect has been discussed elsewhere [74].

A sound experimental foundation may enable the transfer of a suitably modified endosymbiont into diverse crop plants, offering a low cost, environmentally benign source of nitrogen in such a way that might harness the creative input of local farmers and the society the technology is intended to serve.

## Materials and methods

The eight endosymbionts and eight closely related free-living cyanobacteria whose genomes were considered in this study are shown in Fig. 1 with their sources shown in Supplemental Table S1. Broader consideration of cyanobacteria was done through searches of the 127 cyanobacterial genomes within BioBIKE [181] and the 277 semi-curated or highly curated cyanobacterial genomes of CyanoCyc [182], searching by both Blast [183] and built-in orthology functions. In the cases of *Crocosphaera watsonii* WH 8501 and *Rippkaea orientalis* PCC 8801, the genome versions in BioBIKE differ from the most recent versions. The poorly annotated small plasmids of the endosymbionts of *Epithemia adnata* Bon19 and *Rhopalodia gibba* 17Bon1 [38] were not considered in this study, nor were the similar plasmids of endosymbionts of *Epithemia turgida* [35] and *Rhopalodia gibberula* [36]. It is not known whether the other two endosymbionts have plasmids.

Orthologs of proteins were provisionally defined as bidirectional best Blast hits [184]. A bidirectional best hit is one in which Protein A in Organism X best matches Protein B in Organism Y, and Protein B best matches Protein A. Orthologs were generally found through the ORTHOLOGS-OF function of BioBIKE or a similar function in CyanoBIKE. BioBIKE’s function adds an additional restriction that the match must have a better E-value than 10^–10^. The restriction was relaxed to 10^–3^ for proteins whose lengths are less than 100 amino acids. Sometimes orthology was determined manually, particularly when the ORTHOLOGS-OF function was confused by duplicate genes. To obtain the set of core proteins common to free-living cyanobacteria related to the endosymbionts BioBIKE’s COMMON-ORTHOLOGS-OF function was used. For technical reasons, proteins from *Crocosphaera watsonii* WH 8501 were not included in determining common orthologs.

Whenever an ortholog existed to a protein in the carefully annotated *Synechocystis* PCC 6803, the annotation from CyanoCyc was used, noting in Supplemental Table S2A whether the annotation is based on experimental evidence. Otherwise the annotation was from RefSeq [185]. Sometimes, functional annotation was taken from orthologous proteins in heterotrophic bacteria with experimental justification, often found in EcoCyc [186].

The phylogenetic tree of endosymbionts and closely related cyanobacteria was based on concatenated alignments of 29 conserved proteins (Supplemental Table S5), using Clustal W [187] for the alignment, GBlocks [188] to extract informative columns, and PhyML [189] for the final tree. with a model selected by Smart Model Selection [190], LG as the substitution model, and 100 bootstraps. Trees were visualized and manipulated using FigTree 1.4.2 [191]. Quick individual protein trees were made within BioBIKE using its TREE-OF function and neighbor joining.

The metabolic pathways discussed in the Results section, summarized in Fig. 4, listed in Supplemental Table S2, and made visible in Supplemental Figs. S01-S08 were derived from KEGG metabolic maps [192]. Needless to say, they are ultimately arbitrary simplifications of the complex interplay amongst biochemical reactions. Information about transporters was taken largely from the Transporter Classification Database (TCDB) [135].

## Supplementary Information

Supplementary Material 1. Table S1: Genomes used in this study. Table S2: Proteins and RNA related to selected metabolic pathways. Table S3: Sets of orthologs and their intersections. Table S4: Transport proteins. Table S5: Orthologs used to construct genomic phylogenetic tree.

Supplementary Material 2. Maps of pathways with presence of proteins of endosymbionts superimposed. Figure S01: Glycogen metabolism. Figure S02: Glycolysis. Figure S03: Pentose phosphate / Entner-Doudoroff pathways. Figure S04: Tricarboxylic acid cycle. Figure S05: Biotin biosynthesis. Figure S06: Folate and derivatives biosynthesis. Figure S07: Pantothenate /Coenzyme A biosynthesis. Figure S08: Pseudocobalamin biosynthesis. Other Supplementary Figures. Figure S09: Alignment of Small Chlorophyll-α binding-like proteins. Figure S10: Alignment of maltose transporter-like proteins. Figure S11: α-Glucosidase phylogeny.

## Data Availability

No new databases were generated in this study. Analyses were based on publicly available genomes (see Supplemental Table S1 for links to those genomes). Supplemental Tables S2 through S5 provide in a convenient format publicly available data used in this study
