# Supplementary material for: Genomes of N2-fixing endosymbionts of unicellular eukaryotes and host-independence
Source: BMC Genomics. 2026 Feb 5;27:189. doi: 10.1186/s12864-026-12517-0 (PMC12903740; doi:10.1186/s12864-026-12517-0)

Figure S01 – S08: Metabolic maps

Legend

In each of the 21 maps that follow, enzymatic reactions are shown along with indications of the presence or absence of the associated enzymes in *Rippkaea* PCC 8802, in the Rhopalodian endosymbionts, and in the nitroplast of *Braarudosphaera bigelowii* (UCYN-A). The specific genes encoding the relevant proteins are given in Supplemental Table S2, along with the names and symbols of the proteins.

The drawings of the maps are derived -- but highly simplified -- from KEGG metabolic maps [Kanehisa et al, 2021]. Most reactions not present in Rippkaea PCC 8802 have been eliminated. Rarely, reactions indicated by appropriate literature to be pertinent have been added.

The presence or absence of enzymes are indicated in the following ways

- Present in *Rippkaea* PCC 8002, Rhopalodian endosymbionts, and UCYN-A
- * Present in some but not all Rhopalodian endosymbionts
- Present in Rhopalodian endosymbionts but not *Rippkaea* PCC 8002
- Absent in Rhopalodian endosymbionts
- Absent in UCYN-A
- Absent in *Rippkaea* PCC 8002 and all endosymbionts
- Complicated… see comment in Supplemental Table S2 (appropriate tab)

As an additional cue, enzymes present in *Rippkaea* PCC 8002 and the Rhopalodian endosymbionts have names in black font, those absent in the endosymbionts have names in red font, and those present only in the Rhopalodian endosymbionts have names in blue font. Those absent in all considered genomes have names in gray font. The EC numbers in the boxes can serve to identify the enzymes in Supplemental Table S2 (appropriate tab).

Outside pathways that contribute substrates to the pathway under consideration are outlined in pink if the complete pathway is absent in UCYN-A.

See Supplemental Table S1 for the source of genomes and appropriate tabs in Supplemental Table S2 for details about genes and abbreviations in the mapped pathways.

Figure S01: Glycogen metabolism

(derived from KEGG pathway map 00500)

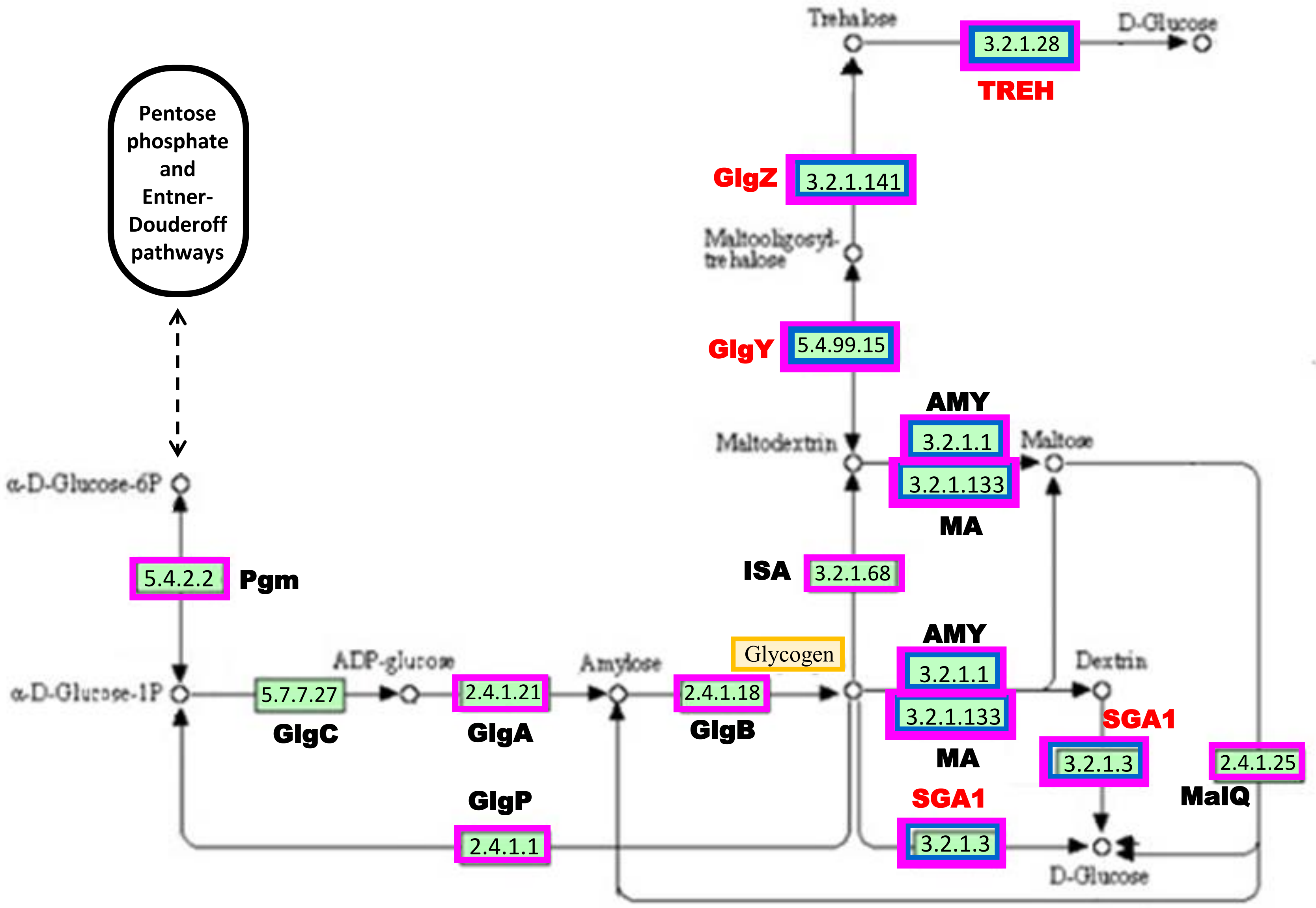

Figure S02: Glycolysis

(derived from KEGG pathway map 00010)

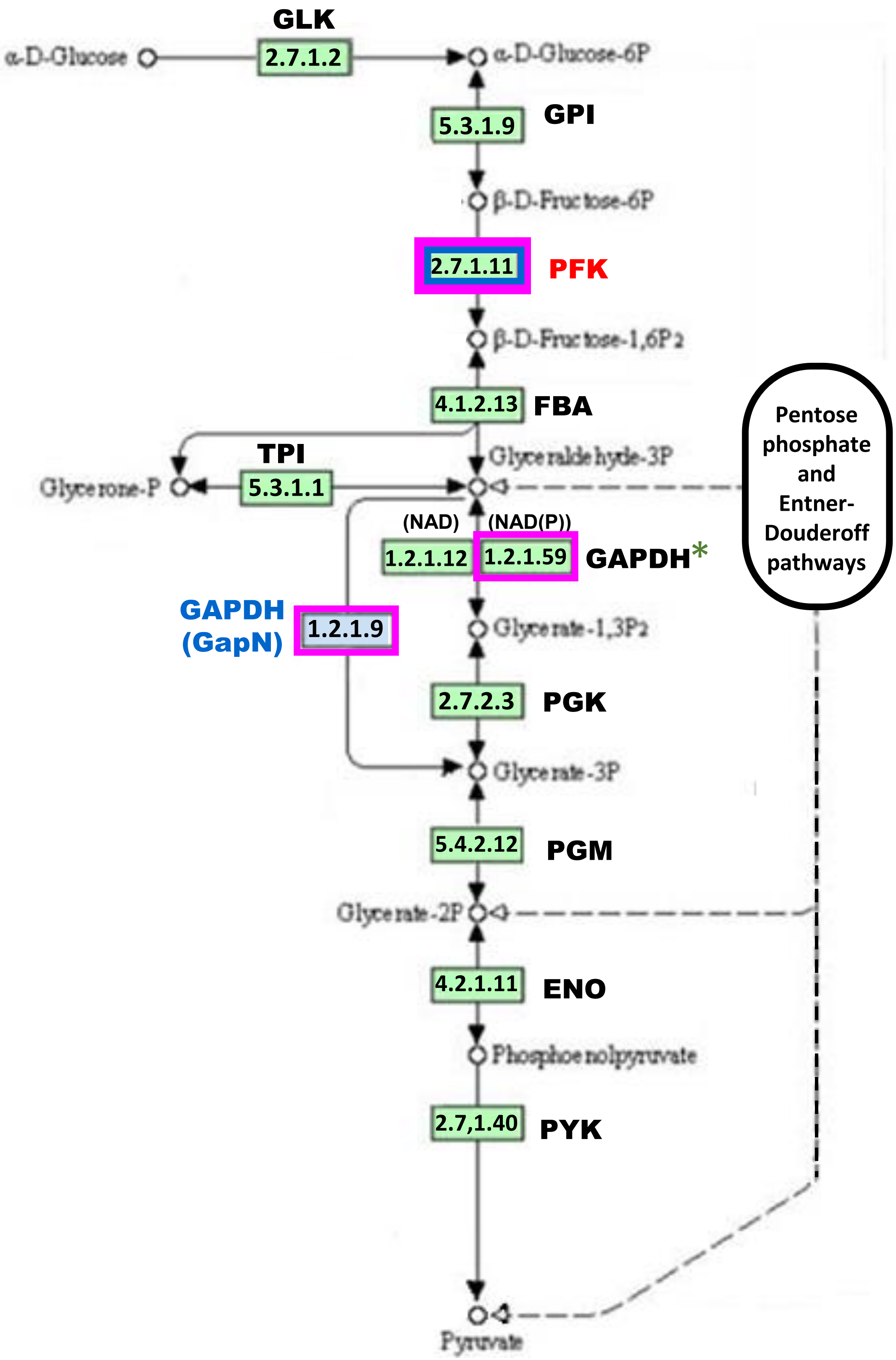

Figure S03: Pentose Phosphate and Entner-Douderoff Pathways

(derived from KEGG pathway map 00030)

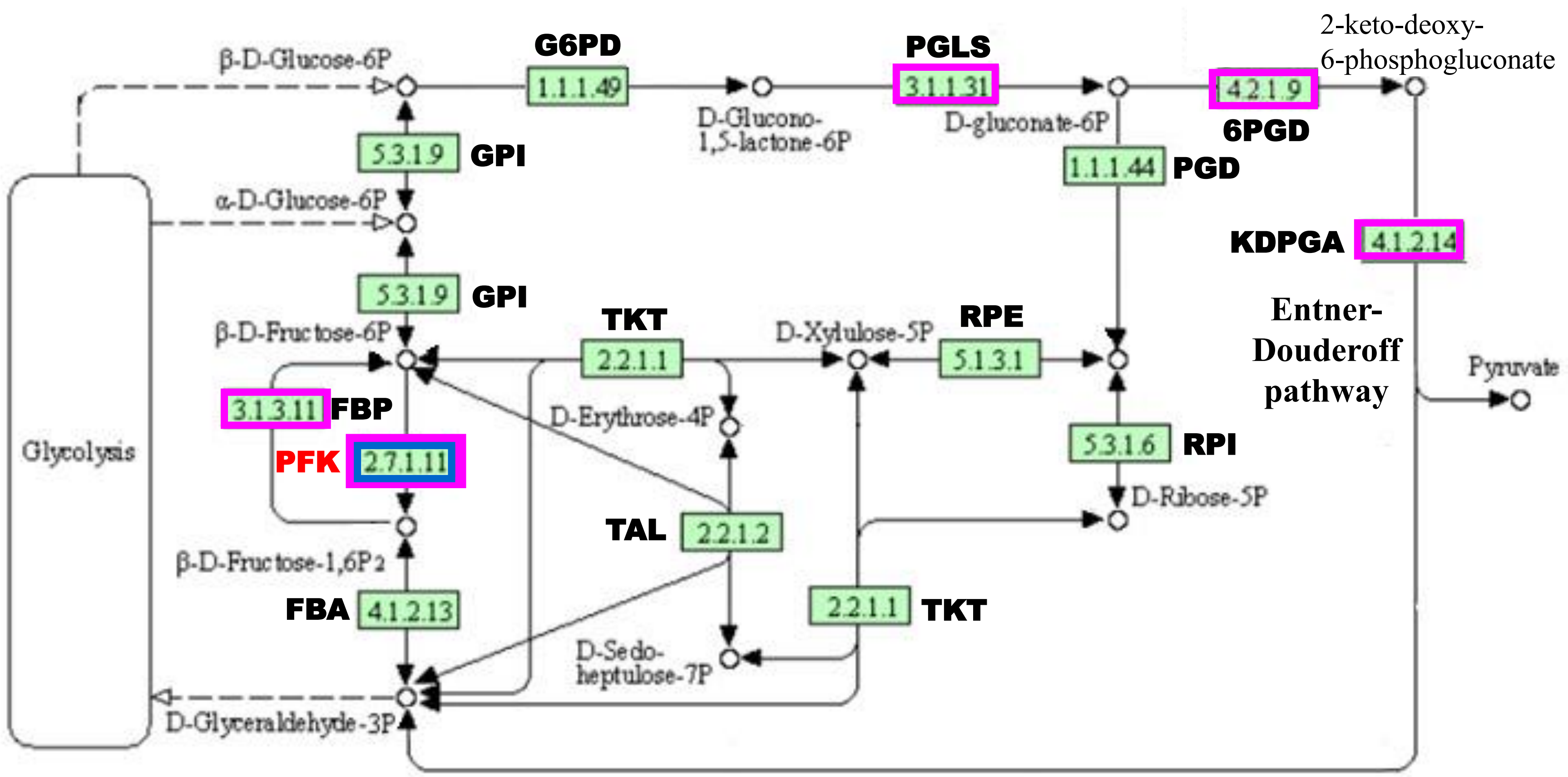

Figure S04: Tricarboxylic acid cycle

(derived from KEGG pathway map 00020)

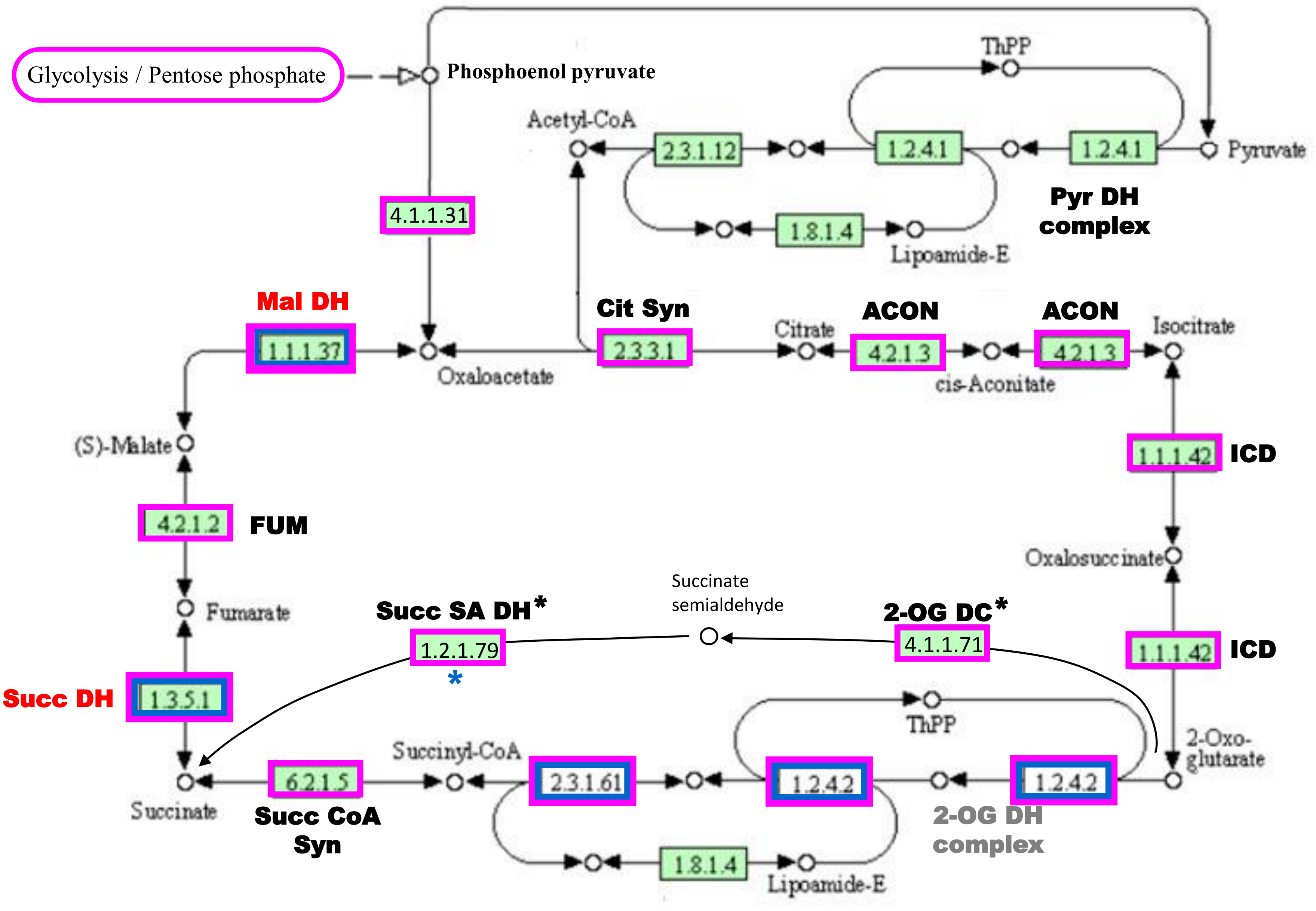

Figure S05: Biotin biosynthesis

(derived from KEGG pathway map 00780)

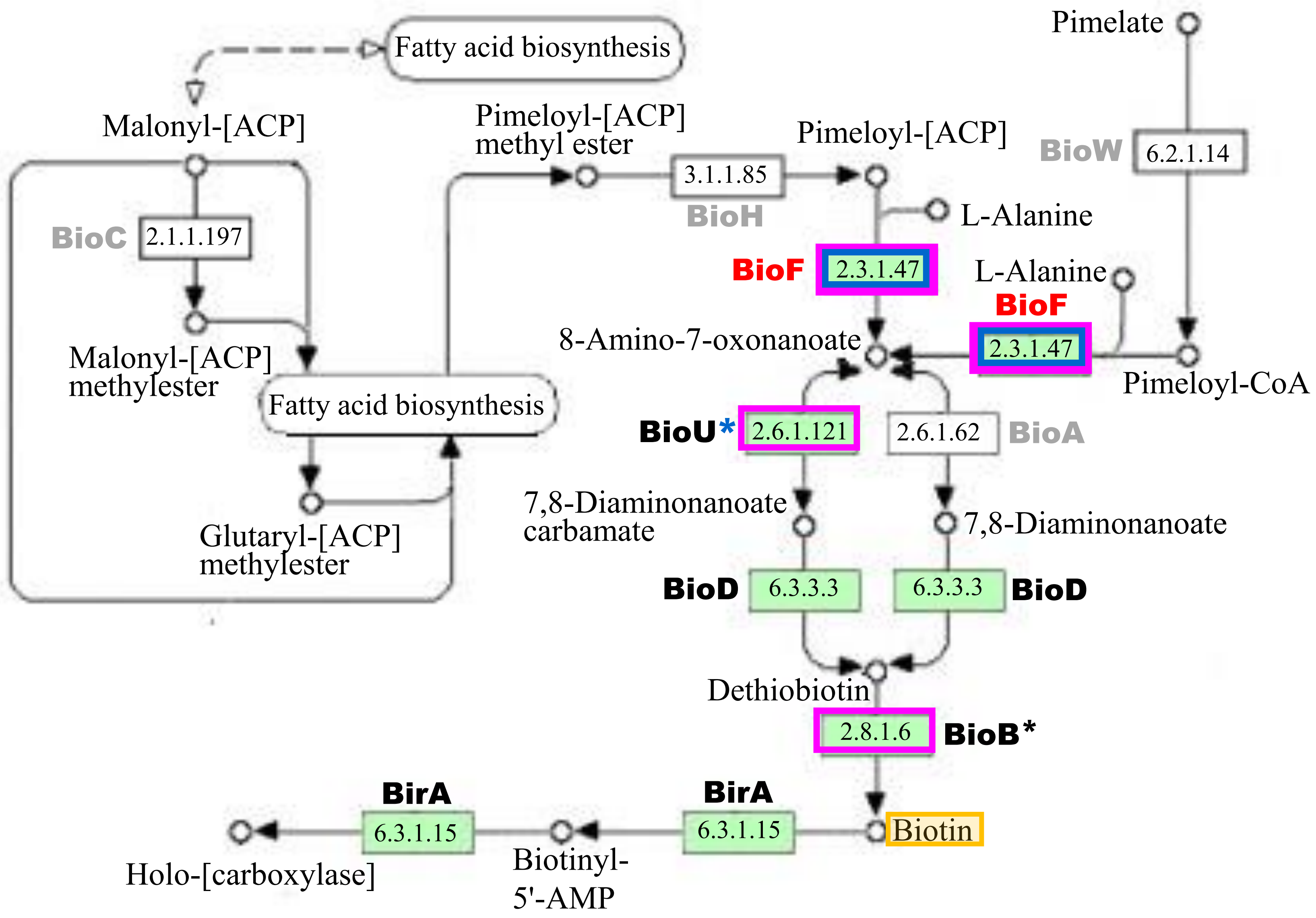

Figure S06: Folate and derivatives biosynthesis

(derived from KEGG pathway map 00790)

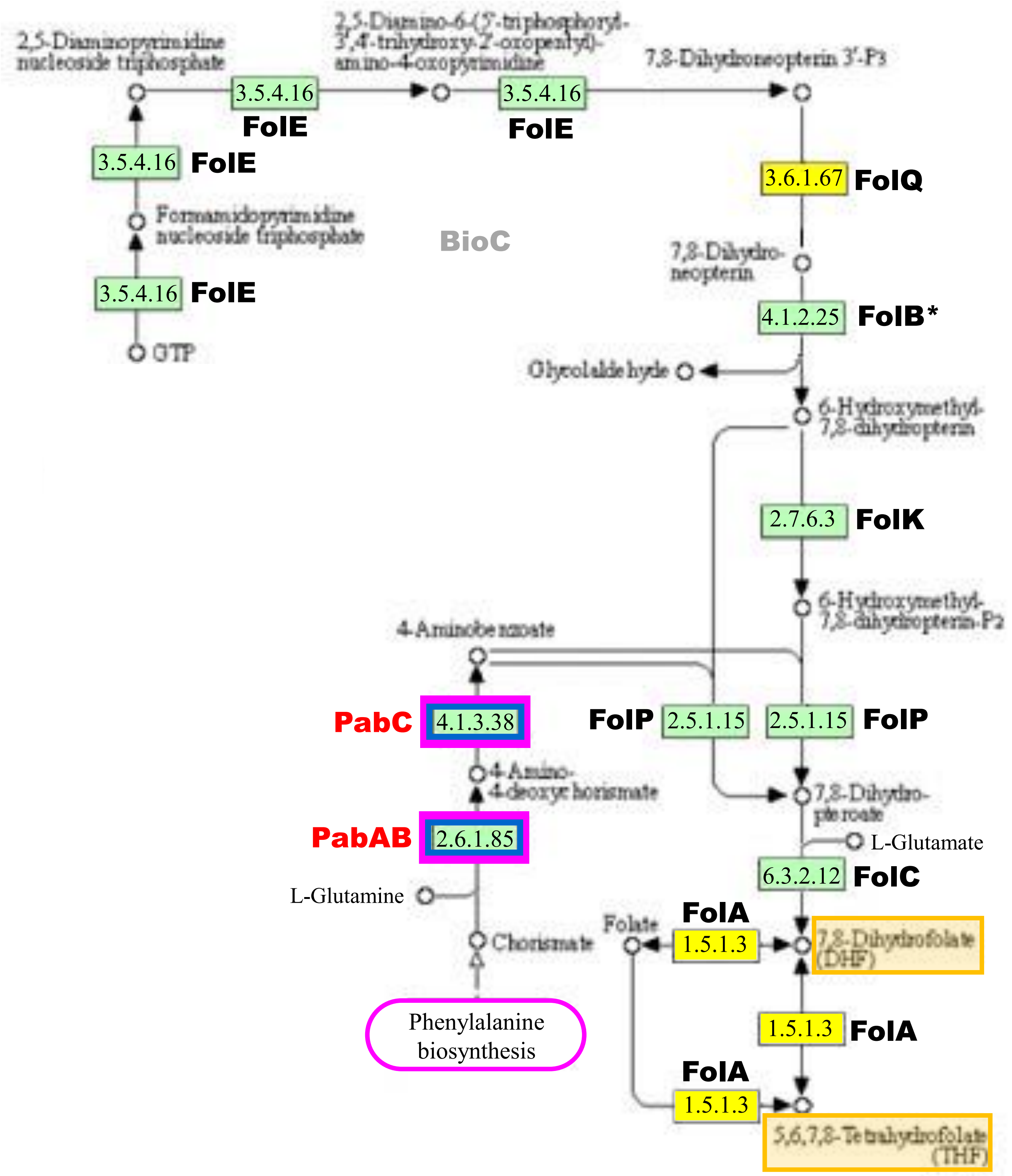

Figure S07: Pantothenate / CoenzymeA biosynthesis

(derived from KEGG pathway map 00770)

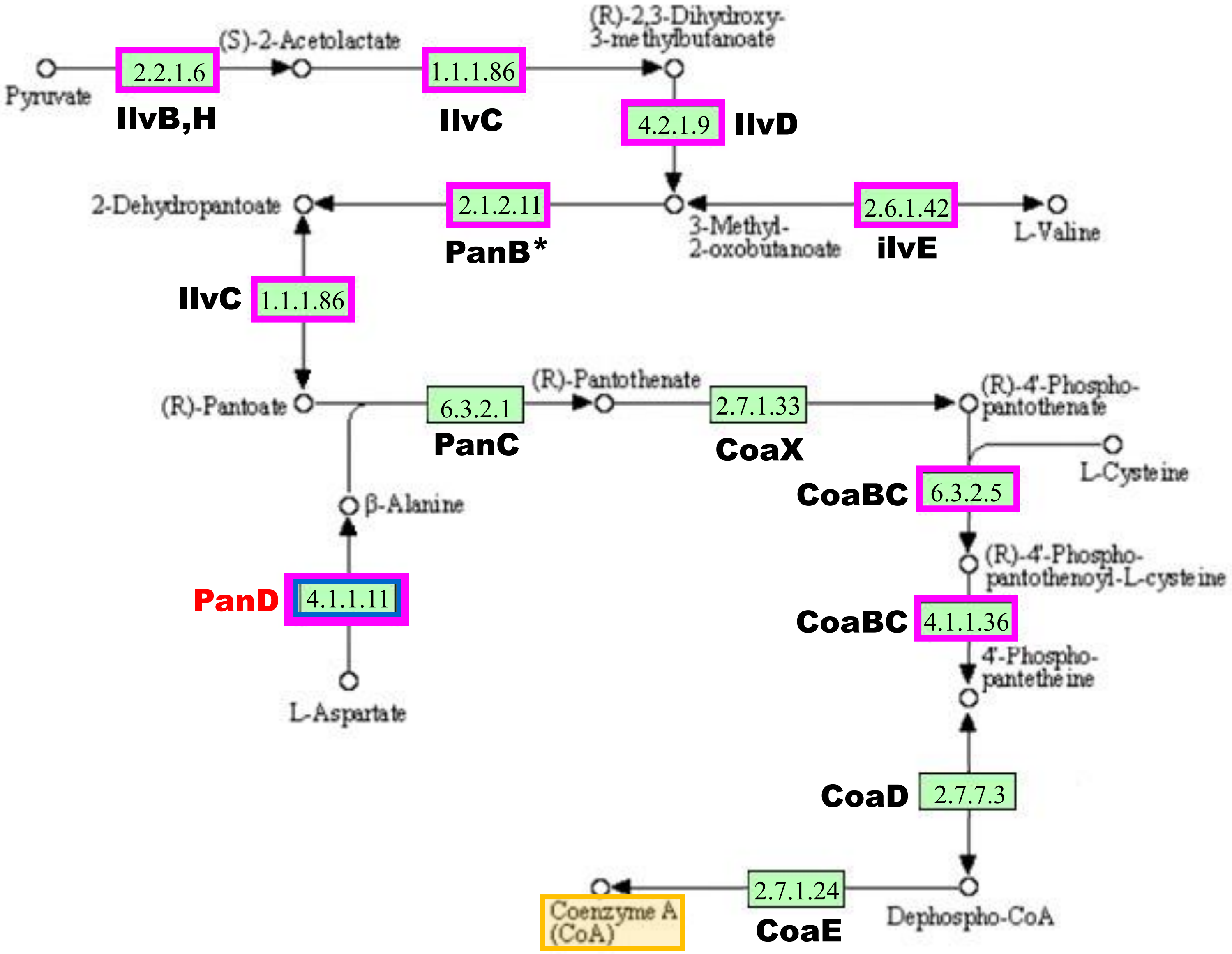

Figure S08: Pseudocobalamin biosynthesis

(derived from KEGG pathway map 00860)

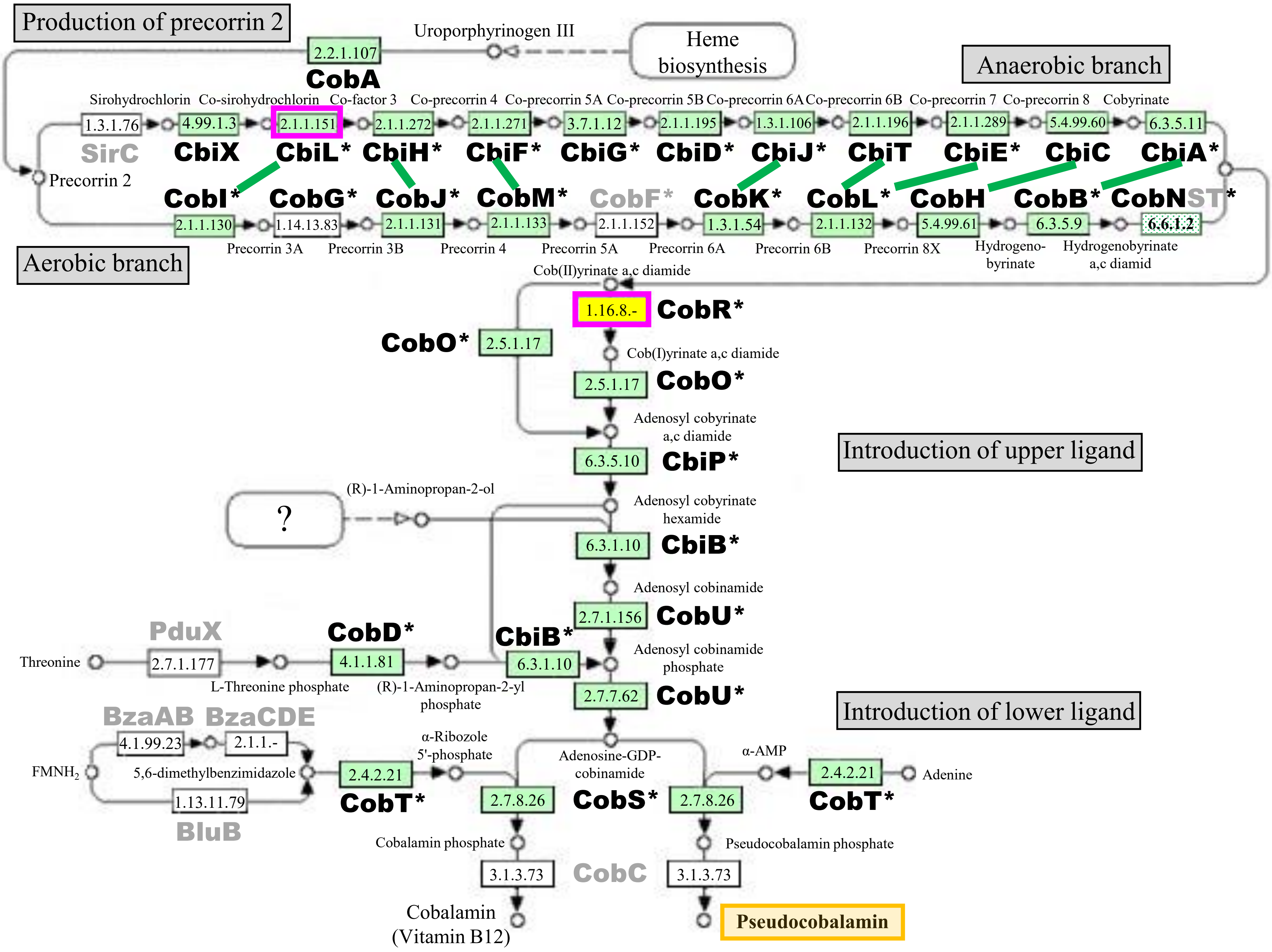

Supplemental Fig. S09: Alignment of Small Chlorophyll-α binding-like proteins*

Symbol	Organism / Protein
**Free-living cyanobacterial relatives of Rhopalodian endosymbionts**	
C51142	Crocosphaera (Cyanothece) ATCC 51142<br>ScpA' (Cce_2568), ScpB (Cce_1543), ScpD (), ScpE (Cce_4826)
R8802	Rippkaea (Cyanothece) PCC 8802<br>ScpA' (Cyan8802-0327), ScoE (Cyan8802-0691)
S6803	Synechocystis (Kazusa) PCC 6803<br>ScpA' (Slr0839), ScpB (Ssl1633), ScpC (Ssl2542), ScpD (Ssr2595), ScpE (Ssr1789)
**Endosymbionts with nearly intact chlorophyll biosynthesis pathways**	
EpSB	Endosymbiont from Epithemia pelagica<br>ScpA' (OXU93-06980)
EcSB	Endosymbiont from Epithemia clementina<br>ScpA' (chromosome from 104894 to 103860), ScpB (P3F56-03335)
RgSB	Endosymbiont from Rhopalodia gibberula<br>ScpA' (RgRSB-0066)
UCYN-A	Endosymbiont from Braarudosphaera bigelowii<br>ScpA' (UcynA-06750), ScpE (UcynA-03290)
**Endosymbionts without nearly intact chlorophyll biosynthesis pathways**	
RaSB	Endosymbiont from Rhopalodia gibba 17Bon1<br>ScpA' (JJP05-05120), ScpE (JJP05-08540)
EaSB	Endosymbiont from Epithemia adnate Bon 19<br>ScpA' (KPI85-04805)
EtSB	Endosymbiont from Epithemia turgida<br>ScpA' (EtSB-0121)

* Residues in alignment highlighted in green match residues identified by Funk & Vermaas (1999). Biochem (1999) 28:9937-9404 conserved amongst all SCP proteins.

Supplemental Fig. S09: ScpA-E alignment (continued)

```
ScpA' (Slr0839)   1 ---------- ---------- ------MDSL NDPPCTFETV PHPKKN-MKM
ScpA' (R8802)     1 ---------- ---------- ------TKSL QEPPCTFNQV IHPKEN-MKM
ScpA' (C51142)    1 AGITNFLRVP ALNTHPLFIN ALADLVTHSL QASPVTFDCV THPKEN-MKM
ScpA' (RgSB)      1 ---------- ---------- ------TKSL QEPPCTFDTV IRPNRN-LKS
ScpA' (EcSB)      1 ---------- ---------- ------TKSL QEPPCTFDSV IRPRKN-LKI
ScpA' (EtSB)      1 ---------- ---------- ------TKSL KEPPCTFDTV IRLKKI--KI
ScpA' (EaSB)      1 ---------- ---------- ------TKSL KEPPCTFDTV IRLKKI--KI
ScpA' (RaSB)      1 ---------- ---------- ------TKSL EEPPCTFDAV IRPKKTPQNI
ScpA' (EpSB)      1 ---------- ---------- ------TKSL QESPYTFDKV IRPKEN-RII
ScpA' (UCYNA)     1 ---------- ---------- ------IHSL EKPPVTFKCI THPREN-III
ScpB  (C51142)    1 ---------- ---------- ---------- ------MKSN NQNEG-----
ScpB  (EcSB)      1 ---------- ---------- ---------- ------MTNS KQYER-----
ScpB  (Ssl1633)   1 ---------- ---------- ---------- ------MNNE NS--------
ScpE  (C51142)    1 ---------- ---------- ---------- -MSEPSTQPT ETPNLE----
ScpE  (R8802)     1 ---------- ---------- ---------- -MADSQSQPT ESPKLE----
ScpE  (Ssr1789)   1 ---------- ---------- ---------- -MSE-ELQPN QTPVQE----
ScpE  (RaSB)      1 ---------- ---------- ---------- -MPDSQFQPT KTPKLR----
ScpE  (UCYNA)     1 ---------- ---------- ---------- -----MNTNT DKRAFD----
ScpD  (Ssr2595)   1 ---------- ---------- ---------- -MTSRGFRLD QDNRLNNFAI
ScpD  (C51142)    1 ---------- ---------- ---------- -MTSRSYVTE EQGRLNNYAI
ScpC  (Ssl2542)   1 ---------- ---------- ---------- -MTTRGFRLD QDNRLNNFAI

ScpA' (Slr0839)  24 YPQ----ERW EWGLTTAAEV WNGRLAMLGF I-ALLVELIS GQGPLHFVGL L
ScpA' (R8802)    24 YPQ----ERW QWGLTTAAEV WNGRLAMVGF I-ALLIELIS GHGPLHFVGL L
ScpA' (C51142)   50 YPQ----ERW QWGMTTAAEV WNGRLAMIGF L-ALLIELIT GYGPLHYVGL L
ScpA' (RgSB)     24 CTQ----KHY ------ILKL FKKLISNIGK L-LKKQELKI FIEFLR---- -
ScpA' (EcSB)     24 YPQ----E-- ---------- ---------- ---------- ---------- -
ScpA' (EtSB)     23 CP-------- ---------- ---------- ---------- ---------- -
ScpA' (EaSB)     23 CP-------- ---------- ---------- ---------- ---------- -
ScpA' (RaSB)     25 TY-------- ---------- ---------- ---------- ---------- -
ScpA' (EpSB)     24 CSQ----EYW RRSITTTIEV WNTRLAIIGF I-ALLFELIS GHGLLHLAGL L
ScpA' (UCYNA)    24 YPK----KSW QWGITSTAEV WNGRLAMLGF V-ALLVELIS GHGILHYLGI I
ScpB  (C51142)   10 ---------- KFGFTGYAEN WNGRLAMIGF ISALIVELVT GQGVLHFWGL L
ScpB  (EcSB)     10 ---------- KFGFTAYAET LNGRLAMIGF LAVLITELVT VQEVLHFWGL L
ScpB  (Ssl1633)   7 ---------- KFGFTAFAEN WNGRLAMIGF SSALILELVS GQGVLHFFGI L
ScpE  (C51142)   16 EP-------- KFGFNDYAER LNGRAAMVGF LLILVIEYLT GQGLLSWLGL Q
ScpE  (R8802)    16 DP-------- KFGFNDYAER LNGRAAMVGF VLTLVIEYLT GQGLLSWLGL H
ScpE  (Ssr1789)  15 DP-------- KFGFNNYAEK LNGRAAMVGF LLILVIEYFT NQGVLAWLGL R
ScpE  (RaSB)     16 EP-------- KFDFNEYTKH LNVRTAIIGF VPSLLIEYLT GQGLLNSLG- -
ScpE  (UCYNA)    12 EP-------- KFGFTEYAER LNGRAAMIGI MSIFIIEYIS GQGIFHWLGL -
ScpD  (Ssr2595)  20 EPPVYVDSSV QAGWTEYAEK MNGRFAMIGF VSLLAMEVIT GHGIVGWLLS L
ScpD  (C51142)  20 EPKMYVDQKK QFGFNKYAEK LNGRLAMIGF ISLLAFESLT GQGLVTWLTN L
ScpC  (Ssl2542)  20 EPEVYVDSSV QAGWTKYAEK MNGRFAMIGF ASLLIMEVVT GHGVIGWLNS L
```

Supplemental Fig. S10: Alignment of maltose transporter-like proteins

Symbol	Organism / Protein	Conserved residues
**Heterotrophic bacteria**		
MalY-CauCr[a]	Caulobacter crescentus (vibrioides) CB15 / Cc2283 (Q9A612)	126
MalT-LacRe[b]	Lactobacillus reuteri TD1 / KEK15710	126
MalT-SheOn[c]	Shewenella oneidensis / WP_011072433	126
**Free-living cyanobacterial relative of Rhopalodian endosymbionts**		
C51142	Crocosphaera (Cyanothece) ATCC 51142 / Cce_4761	93 (74%)
**Rhopalodian endosymbionts with MalT-like proteins**		
EpSB	Endosymbiont from Epithemia pelagica / OXU93-05180	91 (72%)
EcSB	Endosymbiont from Epithemia clementina / P3F56-03655	89 (71%)
RgSB	Endosymbiont from Rhopalodia gibberula / RGRSB-0706	91 (72%)
RaSB	Endosymbiont from Rhopalodia gibba / JJP05-08305	89 (71%)

[a] Described by Lohmiller et al (2008). Microbiol 154:1748-1754.

[b] Described by Zhao & Gänzle (2018). Int J Food Microbiol 272:12-21.

[c] Described by Rodionov et al (2010). BMC Genom 11:494.

```
MalT-LacRe    1 ---------- ---------- ---MSQEKST G-------AG LPTLSKSTIW
MalT-SheOn    1 ---------M SADHTVTQLD S-FSATTHSH ATHS-----V QPELNFWQIF
MalY-CauCr    1 MHFTLRVASA RAQMPPAKRD EKFCEPAGST GGRSGGGSMA RQRLSFLQIW
      RgSb    1 ---------- ---------- -----MEKKF ---------K KKVRNFWQLW
      RaSb    1 ---------- ---------- ----MTEKKI ---------T KKVQNFWQLW
      EcSB    1 ---------- ---------- --------MT ---------E KKIRNFWQLW
      EpSB    1 ---------- ---MIKVKSS QKISYDENKI ---------N KQVQSFWQLW
    C51142    1 ---------- ---------- ---MMTTEKK ---------P NRTRNFFELW
 consensus    1                                                 .   ::

MalT-LacRe   21 MINFGFLGVQ TAFTLQSSQM SRIFQTIGAD PNNLGWFFIL PPLAGLIVQP
MalT-SheOn   36 NMCFGFLGIQ FGFALQNANV SRIFQTLGAS IDEIPILWIA APLTGLLVQP
MalY-CauCr   51 NMCFGFFGIQ IGFGLQNANT SRIFQSLGVD VNHLAILWIA APATGLLVQP
      RgSb   17 NMNVGFFGVQ YGWALQMANT SAIYEYLGAD PEQIPTLWLA APLSGLIAQP
      RaSb   18 NMNVGFCGIQ YGWALQMANT SAIYEYLGAD PEQIPMLWLA APLSGLIAQP
      EcSB   14 NMNVGFFGIQ YGWALQMANT SAIYEYLGAE PEQIPMLWLA APLSGLIAQP
      EpSB   29 NMNVGLFGIQ YGWALQMANT SAIYEYLGAD PEQLPMLWLA APLSGLIAQP
    C51142   19 NMSFGFFGIQ YGWALQMANT SAIYEYLGAN PEQIPLLWLA APVSGLIAQP
 consensus   51  : .*: *:*  .: ** ::  * *:: :*..  :.:  :::  .* :**:.**

MalT-LacRe   71 IIGYYSDRTW APKLGGRRLP YLLLGMIVAV IVMILLPNSG SFGFGYGSLA
MalT-SheOn   86 IIGYLSDNTW G--CLGRRRP YFLIGAILTT LAIFVMPHS- ---------P
MalY-CauCr  101 IIGHFSDKTW G--RFGRRRP YFFWGAILTT LALLVMPNS- ---------P
      RgSb   67 IIGYMSDHTW G--PLGRRRP YFLLGAVISS IALILMPNS- ---------S
      RaSb   68 IIGYMSDRTW G--PLGRRRP YFLLGAILSS IALILMPNS- ---------S
      EcSB   64 IIGYMSDRTW G--PLGRRRP YFLLGAIFSS IALILMPNS- ---------S
      EpSB   79 IIGYMSDRTW G--PLGRRRP YFLLGAILSS IVLVLMPNS- ---------S
    C51142   69 IIGYMSDRTW G--PLGRRRP YFLVGAILSS IALVLMPNS- ---------S
 consensus  101 ***: **.** .    *** * *:: * :.: :.:.::*:*           .
```

Supplemental Fig. S10: Alignment of MalT-like proteins (continued)

```
MalT-LacRe    121 ALWFGAITVA LLDLSSNVAM QPFKMMVGDM VNDDQKSYAY GIQSFLSNTG
MalT-SheOn    124 TLWIAAGMLW IMDASINIAM EPFRAFVGDN LPPSQRTQGY AMQSFFIGIG
MalY-CauCr    139 TLWVAAAALW IMDASINITM EPFRAFVGDN LPDEQRATGY AMQSFFIGLG
      RgSb    105 SLWMAAGLLW ILDTSINISM EPFRAFITDL LPQKQHTRGF SMQSFFIGAG
      RaSb    106 SLWMSAGLLW LLDTSVNISM EPFRAFIVDL LPQKQHTRGF SMQSFFIGAG
      EcSB    102 SLWMAAGLLW ILDTSVNISM EPFRAFIADL LPQKQHTRGF SMQGFFIGAG
      EpSB    117 SLLMAAGLLW ILDTSVNISM EPFRAFITDL LPQKQHIIGF SMQSFFIGAG
    C51142    107 TLWMAAGLLW ILDTSVNISM EPFRAFIADL LPEKQHTQGF SMQTFFIGFG
 consensus    151 :* ..*  :   ::* * *::* :**: :: *   :   .*:   .: .:* *: . *

MalT-LacRe    171 AVLAAVFPFI LTAWFGVRNT AK-RGVVPDS VIIAFYVGAA LLVVTSLFTV
MalT-SheOn    174 AVVASALPYI LSNFFNVANT AP-AGEIADS VRYAFYFGGT VLFLAVTWTV
MalY-CauCr    189 AVFASALPWM LTNWFDVANT AP-AGQVPDS VRIAFYTGGA GLLLAVLWTV
      RgSb    155 AVMASLSPWI LTHLFHVGDR R--DLGVPFS VKLSFYIGAV VFLGTVIWTV
      RaSb    156 AVMASLSPWI LTHLFHLGDK R--EPGVPFS VKLSFYIGAV VFLGTVVWTV
      EcSB    152 AVTASLFPWI LTHLFHVDDR K--ELGVPFS VKSSFYIGAA VFLGTVIWTV
      EpSB    167 AVIASLSPWI LTHIFHMDDT K--KVGIPFS VKSSFYIGAA IFLGTVIWTV
    C51142    157 AVVASVSPWI LTHVFGLSNT TNAAEGVPFT VKVSFYIGAA VFLFTVLWTV
 consensus    201 ** *:   *::  *:   * : :         :. : *   :** *..   :. :   :**

MalT-LacRe    220 FRVHEYDPAT YAKYHGISED DNKEGGNW-- ---------- ----------
MalT-SheOn    223 ISTKEYSPEE LAAFHAKTKT DVEEQCKRSR THKDYQFASF VWMG---LGA
MalY-CauCr    238 FTTREYSPEQ LTAF-EKAER EIAGLGLHEE PEPSVNAYIA LGVGGVLLGA
      RgSb    203 VTTSETPPKN LEKIRNAQKS KSILDKST-- ---------- ----------
      RaSb    204 VTTSETPPKN LEKIQNAQES KSTLNKVT-- ---------- ----------
      EcSB    200 VTTSETPPKN LEKIRNAQKS KSALDKVT-- ---------- ----------
      EpSB    215 VTTSETPPRN LEKIKNFQEG KSTLDKVT-- ---------- ----------
    C51142    207 FTTEEKPPQN LKAMQQANES KDAGDKLG-- ---------- ----------
 consensus    251 . . *   *            :   .

MalT-LacRe    247 ---------- ---------- ---------- ---------- ----------
MalT-SheOn    270 LLTFTVWAQD LDKQLYILSI GIFAFGPLQL YCALRLSQSQ PSQRAQLGMV
MalY-CauCr    287 ALALIVWGAG LEKELYVLAG LLFAFG-LAG VAGARFKRIG RTDNGFS---
      RgSb    230 ---------- ---------- ---------- ---------- ----------
      RaSb    231 ---------- ---------- ---------- ---------- ----------
      EcSB    227 ---------- ---------- ---------- ---------- ----------
      EpSB    242 ---------- ---------- ---------- ---------- ----------
    C51142    234 ---------- ---------- ---------- ---------- ----------
 consensus    301

MalT-LacRe    248 ---FTLLKHA PKAFWTVTLV QFFCWFAFQY LWTYSAGAIA KNVWNTVDAT
MalT-SheOn    320 FNVVDDLFHM PKAMHQLAIV QFFSWFALFA MWIYTTSAVT SYHFGSSDVL
MalY-CauCr    333 -EVLADVFRM PKTMRQLAVV QFFSWFGLFA MWIYTTPAVA TVHFGAVDAS
      RgSb    231 -EILWLIKAM PTTMKQLAVV QFFTWLGIFC VFLYFPPAVA HHIFGAVQEH
      RaSb    232 -EIFWLIKAM PTTMKQLAVV QFFTWLGIFC VFLYFPPSVA HHIFGAVQEN
      EcSB    228 -EIFWLIKAM PTTMKQLAVV QFFTWLGIFC VFLYFPPAVA HNIFGAVREN
      EpSB    243 -EIVLLIKAM PTTMKQLAVV QFFTWLGIFC VFLYFPPAIA HHVFGAVRGD
    C51142    235 -EILNLIKAT PKTMKQLAVV QFFTWLGVFC MFLYFPPAVA HNIFGAVEEN
 consensus    351    .  :     *.::  :::* *** *:..   :: * . :::     :.:
```

Supplemental Fig. S10: Alignment of MalT-like proteins (continued)

```
MalT-LacRe   295 SAGYQAAGNW YGVLSAVQSI AAVIWSYVLA KVPNKY-HKL GYGGSLLLGA
MalT-SheOn   370 SQAYNDGADW VGVLFASYNG FSAIAALFIP LLAKRIGIKL THTFNMFCGG
MalY-CauCr   382 SKAYNEGADW VGVLFAVYNG VAALAALVIP LMVKVTSRRV SHAVCLGLGA
      RgSb   280 SQLYTDGIEW AGICIAVYNG VCFIFSWLLP KLTEIINHKI AHSLCLICGG
      RaSb   281 SQLYTDGIEW AGICIAVYNG ICFIFSLLLP KLTEIINHKI AHSLCLICGG
      EcSB   277 SQLYTDGIEW AGICIAVYNG VCLIFSWLLP KLTEIINHKI AHSLCLICGG
      EpSB   292 SQVYKDGIEW AGICIAVYNG VCIIFSWLLP TLTEIINHKI AHSLCLICGG
    C51142   284 SALYTEGIEW AGICIAVYNG VCFLFSWILP NLTARLGRKM THSLCLICGG
 consensus   401 *  *  . :*  *:  *  .   . : : .:.  :      ::  :   :  *.

MalT-LacRe   344 LGFISVFFVH DQWTLIVSYT LVGIAWAAMN TYPLTIVTNA LTGKHMGTYL
MalT-SheOn   420 FGLISFYFIK DPNLLWLAMI GVGIAWASIL SIPYAILSGT LPPKKMGVYM
MalY-CauCr   432 LGLLSFLVIR DPGLLWIGMV GVGFAWSSIL STPYSILAGA LPARKMGVYM
      RgSb   330 IGLISLVFVD HPLWVLWPMV GFGIAWSSIL AIPYSILSRT LTSQNTGLYM
      RaSb   331 VGLISLVFVE QPLWILLPMV GFGIAWSSIL TIPYSILSRT LTSKNTGLYM
      EcSB   327 VGLISLVFVD QPLLALLPMI GFGIAWSSIL AIPYSILSQT VTSQNIGLYM
      EpSB   342 IGLISLVFVD RPLLVLFPMI GFGIAWSSIL AIPYSILSRT LTGQNTGLYM
    C51142   334 VGLISLLWVN RPIYALFSMV GFGIAWSSTL VIPYSMLSHI IPEKNMGLYM
 consensus   451 .*::*.  :              .*:**::     * ::::   :. :: * *:

MalT-LacRe   394 GLFNGSICLP QIIASLLS-F ALFPLFGHSQ AHMFILAGIV MALGALSVAT
MalT-SheOn   470 GIFNFFIVIP QLLAASVLGL ILNGLFDGQP IYALITGGVF MLCAGIAVLF
MalY-CauCr   482 GIFNVFIVVP QLLAATLLGL MLKTFFGNQS IFALVLGALS FALAAAATFL
      RgSb   380 GIFNAFIVLP QIFAALGLGW IMKIFLESNS LLVVVIGGFS LLLAAVLVHF
      RaSb   381 GIFNVFIVLP QIFAALGLGW IIKIFFKKNC LLVVVLGGFS LLLAAVLVHF
      EcSB   377 GIFNAFIVIP QIFAALGLGW IMKIFLDSNC LLVVVVGGFS LLLAAMLVHF
      EpSB   392 GIFNAFIVLP QIFAALGLGW IMKIFFNSNC LLVVVVGGFS LLLAAVLIYF
    C51142   384 GLFNAFIVIP QIIAALGLGS IMDYFLNNNR LLVVVLGGVS ILLAAICIHW
 consensus   501 *:**  * :* *::*:       :  ::  .     .: ...  :  ..

MalT-LacRe   443 IKETYAE--- ----------
MalT-SheOn   520 VEQPKALTPH ----------
MalY-CauCr   532 VRDRVAGAPA ----------
      RgSb   430 VDEPETLEQT SIQPEILSPS
      RaSb   431 VDEPETSEKT SIQPEILSPS
      EcSB   427 VDKPETSEKT SIQPEILSPS
      EpSB   442 VEEY------ ----------
    C51142   434 VDDIETASTN NQALEVQPTV
 consensus   551 : .
```

Supplemental Fig. S11: α-Glucosidase phylogeny

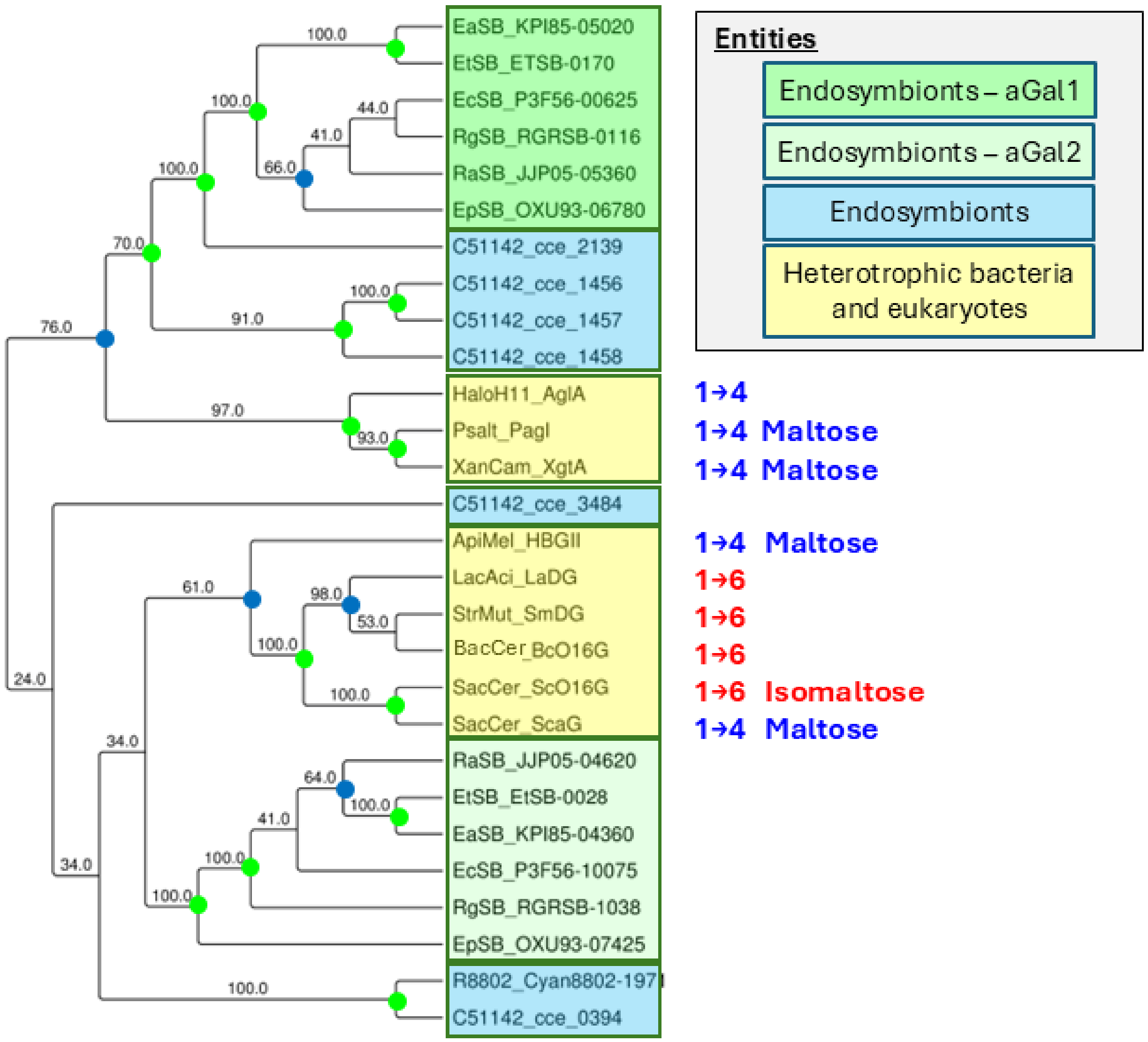

Supplement: Supplementary file 2 — Supplementary Material 2. Maps of pathways with presence of proteins of endosymbionts superimposed. Figure S01: Glycogen metabolism. Figure S02: Glycolysis. Figure S03: Pentose phosphate / Entner-Doudoroff pathways. Figure S04: Tricarboxylic acid cycle. Figure S05: Biotin biosynthesis. Figure S06: Folate and derivatives biosynthesis. Figure S07: Pantothenate /Coenzyme A biosynthesis. Figure S08: Pseudocobalamin biosynthesis. Other Supplementary Figures. Figure S09: Alignment of Small Chlorophyll-α binding-like proteins. Figure S10: Alignment of maltose transporter-like proteins. Figure S11: α-Glucosidase phylogeny. [file 12864_2026_12517_MOESM2_ESM.pdf]
